# Combining Natural Sequence Variation with High Throughput Mutational Data to Reveal Protein Interaction Sites

**DOI:** 10.1371/journal.pgen.1004918

**Published:** 2015-02-11

**Authors:** Daniel Melamed, David L. Young, Christina R. Miller, Stanley Fields

**Affiliations:** 1 Howard Hughes Medical Institute, University of Washington, Seattle, Washington, United States of America; 2 Department of Genome Sciences, University of Washington, Seattle, Washington, United States of America; 3 Department of Medicine, University of Washington, Seattle, Washington, United States of America; The University of North Carolina at Chapel Hill, United States of America

## Abstract

Many protein interactions are conserved among organisms despite changes in the amino acid sequences that comprise their contact sites, a property that has been used to infer the location of these sites from protein homology. In an inter-species complementation experiment, a sequence present in a homologue is substituted into a protein and tested for its ability to support function. Therefore, substitutions that inhibit function can identify interaction sites that changed over evolution. However, most of the sequence differences within a protein family remain unexplored because of the small-scale nature of these complementation approaches. Here we use existing high throughput mutational data on the *in vivo* function of the RRM2 domain of the *Saccharomyces cerevisiae* poly(A)-binding protein, Pab1, to analyze its sites of interaction. Of 197 single amino acid differences in 52 Pab1 homologues, 17 reduce the function of Pab1 when substituted into the yeast protein. The majority of these deleterious mutations interfere with the binding of the RRM2 domain to eIF4G1 and eIF4G2, isoforms of a translation initiation factor. A large-scale mutational analysis of the RRM2 domain in a two-hybrid assay for eIF4G1 binding supports these findings and identifies peripheral residues that make a smaller contribution to eIF4G1 binding. Three single amino acid substitutions in yeast Pab1 corresponding to residues from the human orthologue are deleterious and eliminate binding to the yeast eIF4G isoforms. We create a triple mutant that carries these substitutions and other humanizing substitutions that collectively support a switch in binding specificity of RRM2 from the yeast eIF4G1 to its human orthologue. Finally, we map other deleterious substitutions in Pab1 to inter-domain (RRM2–RRM1) or protein-RNA (RRM2–poly(A)) interaction sites. Thus, the combined approach of large-scale mutational data and evolutionary conservation can be used to characterize interaction sites at single amino acid resolution.

## Author Summary

The interactions of proteins with each other are essential for almost all biological processes. Many of the sites of protein contact have evolved to maintain these interactions, but use different sets of amino acid residues. As a result, the residues at a contact site in a protein from one species might not allow a protein interaction when they are tested in a second species. This property underlies the idea of inter-species complementation assays, which test the effect of replacing protein segments from one species by their equivalents from another species. However, this approach has been highly limited in the number of changes that could be analyzed in a single study. Here, we present a novel approach that combines a high-throughput analysis of mutations in a single protein with the set of natural sequences corresponding to evolutionarily divergent variants of this protein. This integration step allows us to map at high resolution both sites of inter-protein interaction as well as intra-protein interaction. Our approach can be used with proteins that have limited functional and structural data, and it can be applied to improve the performance of computational tools that use sequence homology to predict function.

## Introduction

Protein activity, folding and stability are regulated by the interactions of proteins with other macromolecules. Thus, the identification of sites on a protein where these interactions occur is a critical but difficult undertaking. In some cases, structural analyses provide these sites at high resolution. In other cases, combinations of biochemical, biophysical and genetic methods with mutagenesis strategies have delineated specific residues that contribute to physical interactions. However, the vast number of protein-protein interactions and the low throughput and robustness of approaches to identify interaction sites have led to the limited and often imprecise characterization of only a tiny fraction of the contact sites. Sequence-based computational methods offer an alternative and cost-effective approach that can predict interacting positions by making use of homologous sequences. For example, the evolutionary trace method [1] assumes that the locations of interaction sites are conserved over evolution, and that sequence variation within these sites occurs in response to changes in evolutionary constraints to allow the protein to maintain its activity. Other computational methods are based on the idea that physical interaction between two proteins leads to linked evolutionary changes between their contact sites [2,3,4]. Thus, the correlated changes between pairs of positions in multiple sequence alignments of two interacting proteins can identify binding sites [2]. However, despite improvements in the construction of multiple sequence alignments and phylogenetic trees, and the huge increase in the number of homologous sequences, the accuracy of these methods remains challenged by fundamental problems [5,6]. For example, transient interactions often yield poor evolutionary signals due to increased rates of substitutions at contact sites [7]. In consequence, these contact sites resemble other, less critical residues in the protein that also tolerate multiple substitutions.

We begin with the idea that substitutions tolerated in nature usually cause only minor changes in structure [8]. Thus, if a position in a protein is substituted with an amino acid that is found at that position in homologous proteins, the resulting protein is likely still to function in its native organism. However, when such a substitution has a detrimental effect, it may have affected a functional site that has changed over evolution [9]. For a protein contact site, such a detrimental effect is likely due to the lack of other compensating substitutions also present in the homologous protein that have co-evolved to support its binding to a partner protein. Alternatively, compensatory substitutions might be present in the homologue of the protein partner. Complementation assays using a protein with such natural substitutions have been used to characterize binding site residues [10,11,12,13]. However, the utility of this approach has been limited by the lack of large-scale assays that can test a protein’s activity when it carries all the possible substitutions that occur in homologous sequences.

Recently, a method known as deep mutational scanning was developed to assess the functional consequences of up to hundreds of thousands of variants of a protein in a single experiment [14,15]. This method combines next generation sequencing with a functional selection, using the change in frequency for each variant over the course of the selection as a proxy for the variant’s activity. We previously applied this method to study the *in vivo* function of an RNA recognition motif (RRM) of the *Saccharomyces cerevisiae* poly(A)-binding protein, Pab1 [16].

The eukaryotic poly(A)-binding protein regulates mRNA translation and decay [17,18,19] by binding to the poly(A) tail of an mRNA via its four RRMs [20,21]. This binding leads to an interaction between RRM2 and the translation initiation factor eIF4G, a constituent of the mRNA cap-binding complex, eIF4F [22], which is assumed to enhance the rate of translation by supporting the establishment of a closed loop structure of the mRNA [23,24,25]. Yeast encode two eIF4G paralogues, eIF4G1 and eIF4G2 [26], which both interact with Pab1 [12]. Complementation assays by Otero et al. [12] with yeast Pab1 containing residues from the human orthologue mapped the binding site for the two eIF4G isoforms to five amino acids on the surface of Pab1 RRM2 [12]. However, this study addressed only the 25 Pab1 residues in the RRM2 domain that vary between human and yeast, and thus the contribution of the other 50 RRM2 residues and the precise Pab1 contact sites for the two isoforms of eIF4G were not determined.

We analyzed deep mutational scanning data for the RRM2 domain of yeast Pab1 to examine the functional consequences in yeast of single amino acid substitutions that differentiate the yeast domain from its homologues. This large-scale inter-species complementation data allowed us to characterize the eIF4G1 and eIF4G2 binding sites on the RRM2 surface at single amino acid resolution and to identify residues associated with the RRM2–poly(A) and RRM2–RRM1 interactions. By combining epistasis data for double mutants with natural variation data, we identify a humanizing substitution that promotes a change in binding specificity of the yeast Pab1 RRM2 from the yeast to the human eIF4G1 protein. Taken together, *in vivo* deep mutational scanning data integrated with evolutionary variation can be used to characterize interaction sites with high resolution and to predict epistatically interacting residues in natural homologues of a protein.

## Results

### Effects of substituting amino acids in Pab1 with those of Pab1 homologues

We recently scored the *in vivo* function of more than 100,000 variants of the RRM2 domain of the yeast Pab1 [16]. The assay was based on turning off the expression of a wild-type copy of the *PAB1* coding sequence and assaying growth of yeast dependent on mutated versions of a C-terminally truncated form (Pab1-343) that includes the first three RRM domains and a small portion of RRM4. For each variant, we assigned an enrichment score that represents the ratio between the fractions of its sequence read counts after and before selection, normalized to the wild-type enrichment score. Hence, enrichment scores serve as indirect readouts for the effects of mutations on growth rate. We obtained scores for 1246 single amino acid substitutions, including 1190 missense mutations and 56 nonsense mutations (∼83% of all possible single amino acid substitutions in the 75 amino-acid long sequence that covers most of this domain) [16].

**Figure 1:**
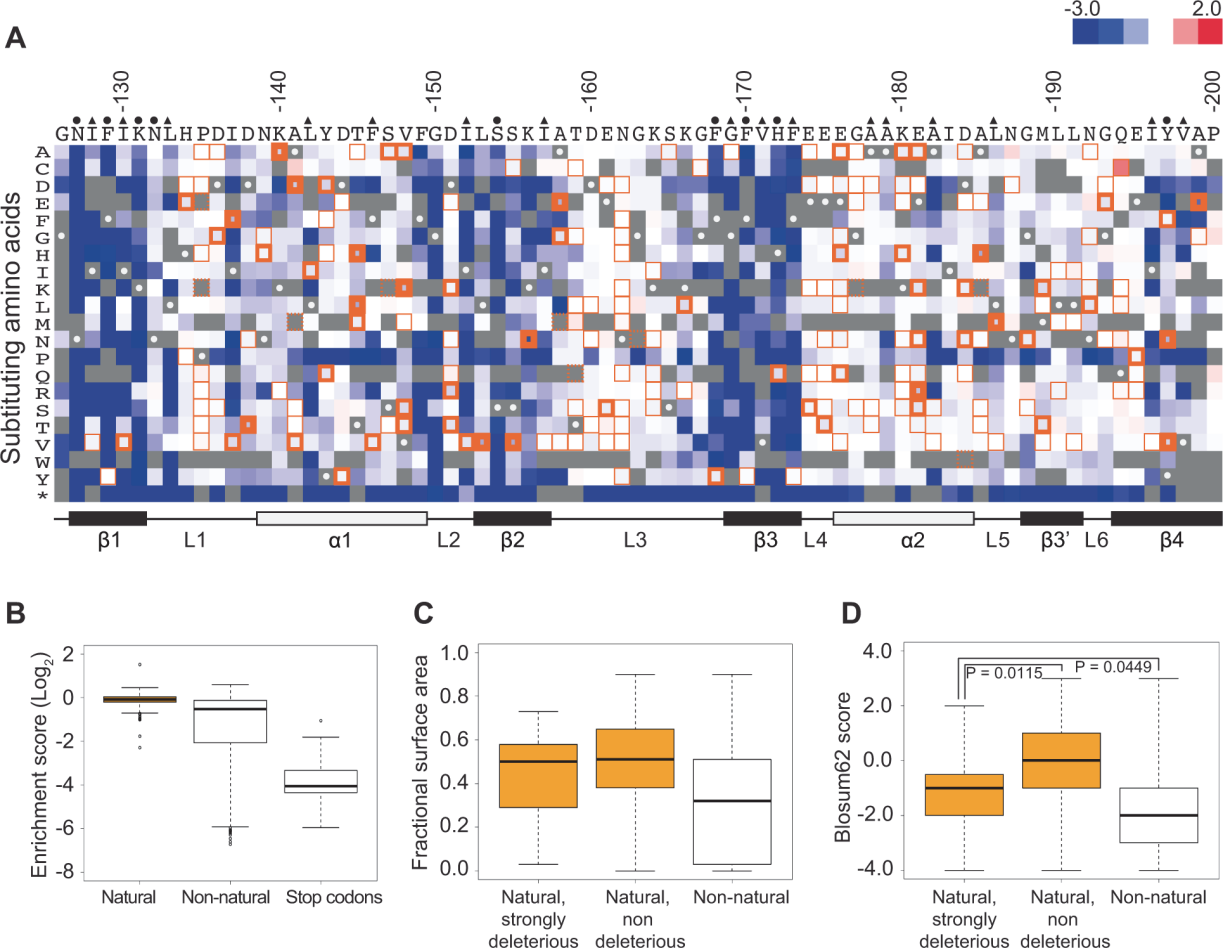
Functional characterization of single amino acid substitutions occurring in 52 Pab1 RRM2 homologues. A) A heat map displaying the enrichment scores (log_2_ transformed) for single amino acid substitutions in the Pab1 RRM2 sequence [16]. Each column represents a site in the RRM2 sequence and each row a substitution to a specific amino acid. An asterisk designates nonsense mutations. Color ranges from blue for the most detrimental mutations to red for the most beneficial. Orange outlines depict substitutions found in at least one of the 52 Pab1 homologues that were analyzed, with effects that are non-deleterious indicated by thin lines, effects that are mildly deleterious by intermediate lines, and effects that are strongly deleterious by thick lines. Gray stands for missing data and substitutions that did not pass the 40 input read counts cutoff or other quality filtration steps. Wild-type residues are marked by gray with white central dots. Residues known to interact with poly(A) in the human RRM2 domain [30] are marked with green and residues showing low solvent accessibility (PDB ID 2K8G, fractional accessible surface area ≤ 0.1) are marked with magenta. The secondary structure of the RRM2 domain aligned to the sequence is shown below. B) The distribution of enrichment scores for amino acid substitutions found in Pab1 RRM2 homologues (natural) and for all other substitutions that do not occur in those sequences (non-natural). Shown also is the distribution of stop codons to highlight enrichment scores of null mutations. C) Box plots displaying the fractional surface area for the equivalent yeast residues in the human RRM2 structure in complex with poly(A) (PDB ID 1CVJ). Shown are the fractional surface area for the 17 amino acid substitutions in Pab1 RRM2 homologues that displayed enrichment scores lower than −0.5, the other naturally occurring substitutions, and the non-natural substitutions. D) Box plots depicting the Blosum62 score of the same substitution groups as in C. p-values from Wilcoxon rank sum tests for the differences between the score distributions are shown.

We realized that the scores of variants with amino acid substitutions present in Pab1 homologues might provide insight into functional sites that diverged in sequence throughout the evolution of this protein. To this end, we collected sequences of 52 poly(A)-binding proteins that represent all Pab1 homologues in the UniProtKB/Swiss-Prot database. The 52 homologous sequences include both orthologues and paralogues of the poly(A)-binding protein and are derived from eukaryotic species including fungi, plants and mammals. All 52 proteins carry four tandem RRM domains, allowing us to align the Pab1 RRM2 against all its corresponding domains. The multiple sequence alignment showed conservation between the homologous RRM2 sequences and the yeast Pab1 RRM2 ranging from 88% identity for *Candida glabrata* to 55% identity for *Encephalitozoon cuniculi*. The alignment revealed 210 single amino acid differences (“natural substitutions”) with respect to the yeast Pab1 RRM2 sequence. The *in vivo* deep mutational scanning data from our previous study [16] provide functional scores for 197 of these 210 substitutions (Fig. 1A).

Most of these natural substitutions resulted in small effects (Fig. 1B), with a median score of −0.07 relative to the wild-type (the score, in log_2_ scale, is comparable to ∼5% reduction from the wild-type score) and narrow upper and lower quartiles. On the contrary, substitutions that do not appear in Pab1 homologues (“non-natural substitutions”) displayed a much larger range and more negative effects, with a median score of −0.53 (comparable to ∼30% reduction from the wild-type score). That most natural changes result in small effects suggests that the functional constraints on the poly(A)-binding protein remained largely constant throughout its evolution. However, a few natural substitutions showed low enrichment scores that correspond to poor Pab1 performance in *S. cerevisiae*. In particular, enrichment scores of 45 natural substitutions ranged between −0.15 and −0.5 (a range that we term mildly deleterious, comparable to ∼10–30% reduction from the wild-type score) and enrichment scores of 17 other natural substitutions were lower than −0.5 (a range that we term strongly deleterious, comparable to more than 30% reduction from the wild-type score) (Fig. 1A). We further compared the score distribution of natural variants to the score distribution of synonymous variants which serve as a proxy for non-deleterious variants, as previously described [16]. This comparison allowed us to assess the contamination of the mildly and the strongly deleterious groups by variants that carry non-deleterious mutations (S1 Fig.). Based on this analysis, we estimated that the natural substitutions in the mildly deleterious group are contaminated by 35% non-deleterious variants, while the natural substitutions in the strongly deleterious group are contaminated by only 8% non-deleterious variants. Given these results, we further analyzed only mutations classified as strongly deleterious.

The solvent accessibility of residues in the structure of a human orthologue of Pab1 reveals that both natural non-deleterious and natural strongly deleterious substitutions, compared to all other non-natural substitutions, occur preferentially at solvent-exposed areas (Fig. 1C). However, an evaluation of the conservation of each substitution using its Blosum62 score revealed a significant difference between the natural non-deleterious and the natural strongly deleterious groups (Fig. 1D). Though both groups showed high conservation compared to non-natural substitutions, the natural strongly deleterious substitutions displayed a lower conservation score (median of −1) than the natural non-deleterious substitutions (median of 0). The differences in Blosum62 score distributions of the two groups suggests that natural deleterious effects in general are due to substitutions to amino acids that display physicochemical properties that are neither as disruptive as non-natural substitutions nor as subtle as natural non-deleterious ones. Nonetheless, a few natural-deleterious substitutions resulted from replacements by highly similar amino acids (*e.g.* L186M and L153V), indicating that sometimes the exact identity of the Pab1 residue is of crucial importance.

### Delineation of the eIF4G-binding site in Pab1

**Figure 2:**
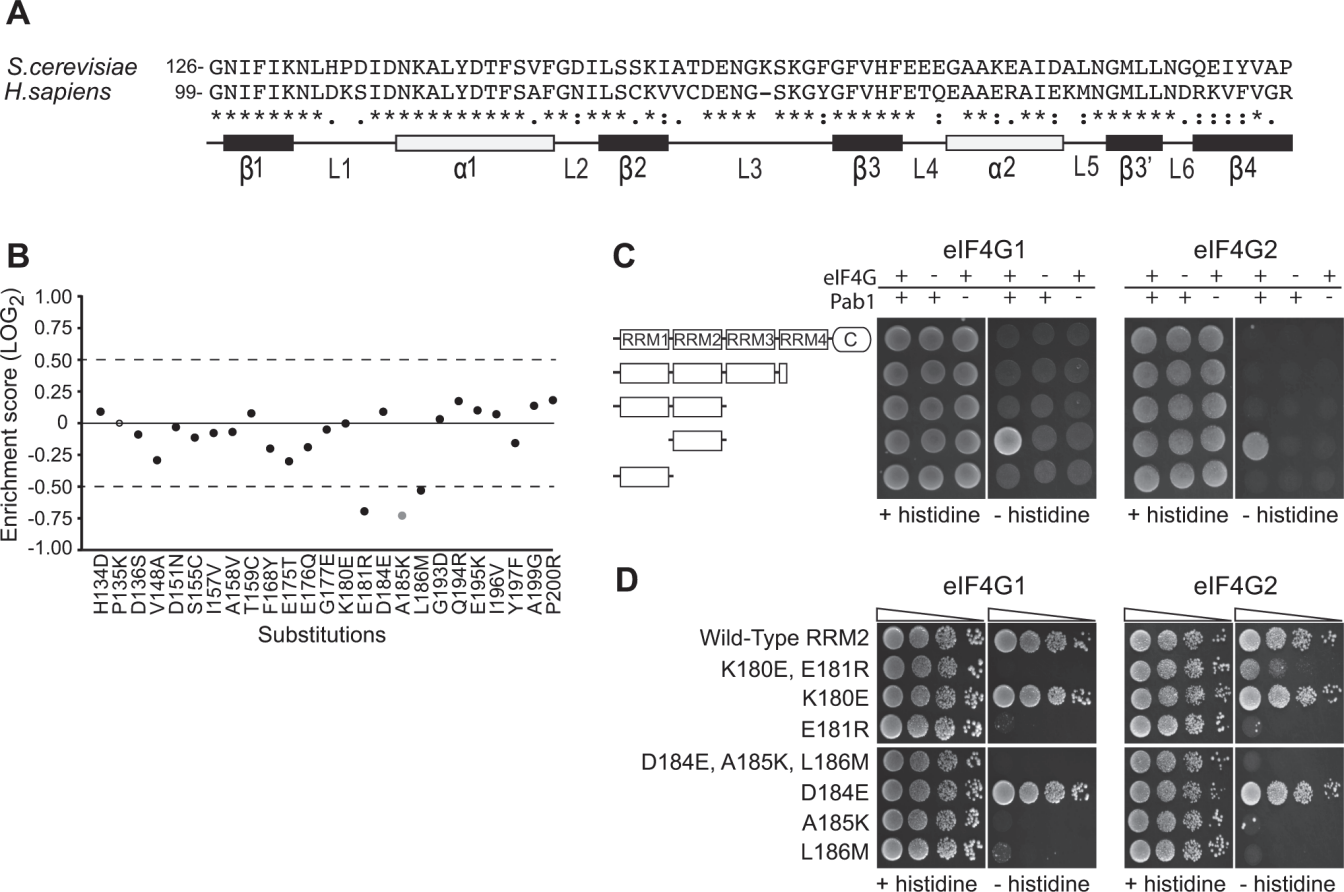
Functional consequences of single amino acid substitutions corresponding to residues found in the human RRM2 domain. A) Sequence alignment of the *S. cerevisiae* Pab1 RRM2 region that was subjected to deep mutational scanning [16] and its human homologue. The illustration below depicts the secondary structure of the human RRM2 domain (based on PDB ID 1CVJ). Black and white rectangles represent β-strands and α-helices, respectively. Lines represent loop structures. B) Enrichment scores for 24 of the 25 single amino acid substitutions that differentiate the yeast RRM2 from its human orthologue. Scores were normalized to the enrichment score of the wild-type protein (black solid line). Black circles represent scores obtained for variants with more than 40 sequencing reads in the input library, which served as a cutoff for reliable measurements [16]. The gray circle represents a less reliable score due to lower input read count. The empty circle stands for missing data. C) A yeast two-hybrid assay testing for the interaction between full length or C-terminal truncations of Pab1 (fused to the Gal4 DNA-binding domain) and the N-termini (amino acids 1–341) of the two eIF4G isoforms (fused to the Gal4 activation domain). For *HIS3* expression, 2*10^4^ cells were seeded on synthetic complete plates selecting only for the two plasmid markers (no histidine selection) or for the two-hybrid interaction (histidine selection), and grown for three days. D) The same eIF4G isoforms were tested against Pab1 RRM2 variants that carry the specified mutations, as described in C. Triangles represent 10-fold dilutions in the number of cells at each spot, beginning with 2*10^4^ cells at the leftmost spots.

Of the 25 single amino acid substitutions that differentiate the yeast Pab1 RRM2 domain from its human orthologue (Fig. 2A), 24 have enrichment scores in our dataset. Three of these mutations (E181R, A185K and L186M) are strongly deleterious (Fig. 2B). These three substitutions occur in two short stretches of the yeast Pab1, 180-KE-181 and 184-DAL-186, that when replaced with the corresponding human stretches to create 180-ER-181 and 184-EKM-186 interfere with *in vitro* binding to ∼100 amino acid fragments of yeast eIF4G1 and eIF4G2 [12,22]. The large-scale mutational data indicate that the other two mutations in these short stretches, K180E and D184E, cause no measurable effect on function (Fig. 2B).

To test whether the *in vivo* effects on Pab1 performance correlate with eIF4G1 and eIF4G2 binding, we established a two-hybrid assay between yeast Pab1 and the N-terminal 341 amino acids of yeast eIF4G1 or eIF4G2, which contain the binding sites for Pab1 [12,22]. The full-length Pab1 tested with the eIF4G1 or eIF4G2 fragment did not activate *HIS3* reporter gene expression (Fig. 2C). However, as some protein-protein interactions can be detected by the yeast two-hybrid system only when parts of the proteins are removed [27], we tested various truncation products of Pab1 for eIF4G1 and eIF4G2 association. Indeed, RRM2 alone produced a positive interaction signal with both isoforms (Fig. 2C).

In agreement with Otero et al. [12], the replacement of residues 184–186 with those from human resulted in complete loss of binding to both eIF4G1 and eIF4G2 (Fig. 2D). When tested individually, A185K and L186M did not bind eIF4G1 or eIF4G2, while D184E showed wild-type binding. The replacement of residues 180–181 with those from human abolished eIF4G1 binding and reduced eIF4G2 binding. This residual binding to eIF4G2 may reflect the greater sensitivity of the two-hybrid assay compared to the *in vitro* assay [12]. When tested individually, E181R resulted in loss of eIF4G1 and eIF4G2 binding, while K180E had no effect (Fig. 2D). Since the E181R effect on eIF4G2 binding was more severe in the absence of the K180E substitution, K180E might suppress the negative effect of the E181R mutation on eIF4G2 binding by decreasing the local positive charge. Overall, the *in vivo* function of Pab1 carrying any of the five single amino acid substitutions correlates with the ability of Pab1 to support eIF4G1 and eIF4G2 binding.

**Figure 3:**
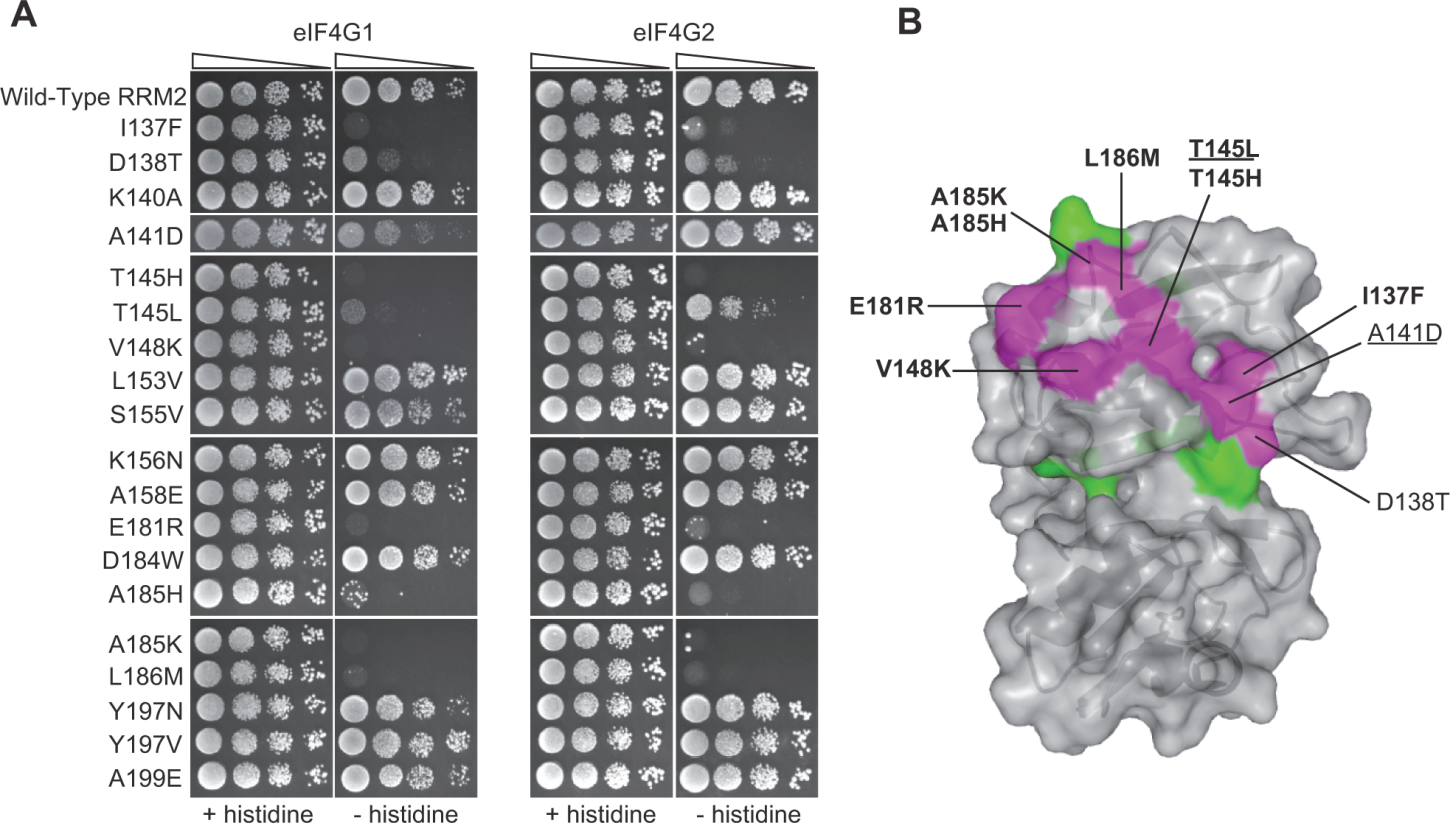
Effects of deleterious substitutions found in Pab1 RRM2 homologues on Pab1 binding to eIF4G. A) A yeast two-hybrid assay testing 19 deleterious substitutions found in Pab1 RRM2 homologues for the ability of Pab1 to bind to the two eIF4G isoforms. The *HIS3* assays were performed as described in Fig. 2. The two substitutions marked with gray did not pass the input read cutoff and therefore their low enrichment score is less reliable. B) Structure of the human orthologue of the Pab1 RRM1-RRM2 fragment bound to poly(A) (PDB ID 1CVJ). Shown is the surface at the opposite side of the RNA-binding site and the secondary structure depicted by a cartoon. Residues with mutations that impaired eIF4G1 or eIF4G2 binding are shown in purple with the substitution names specified. Bold and non-bold markings represent no growth and intermediate growth of mutants under selection. Substitutions that are underlined depict stronger effect on eIF4G1 binding over eIF4G2. Residues with mutations that did not interfere with eIF4G1 or eIF4G2 binding are shown in green.

We hypothesized that the deleterious effects of some of the other natural substitutions might be due to a loss of eIF4G1 and eIF4G2 binding. We therefore tested in the two-hybrid assay the 17 substitutions that cause a strongly deleterious effect, as well as A185K and D184W, which score similarly but had lower sequence read coverage in the original experiments [16]. Of these 19 mutations, 10 (occurring in 8 different residues) impaired the ability of RRM2 to bind eIF4G1, with I137F, T145H, T145L, V148K, E181R, A185H, A185K and L186M showing the most severe effects (Fig. 3A, left). D138T and A141D resulted in modest effects on eIF4G1 binding (Fig. 3A, left). The same Pab1 variants assayed against eIF4G2 revealed similar effects (Fig. 3A, right), suggesting that eIF4G1 and eIF4G2 use the same set of Pab1 RRM2 residues for binding. However, eIF4G1 binding was more sensitive to A141D and T145L compared to eIF4G2. Based on the effects of the natural amino acid substitutions on binding, we set the boundaries of eIF4G recognition site to the upper surface of RRM2 (Fig. 3B), a region much wider than previously identified [12].

### A large-scale mutational analysis of the Pab1 RRM2-eIF4G1 interaction

**Figure 4:**
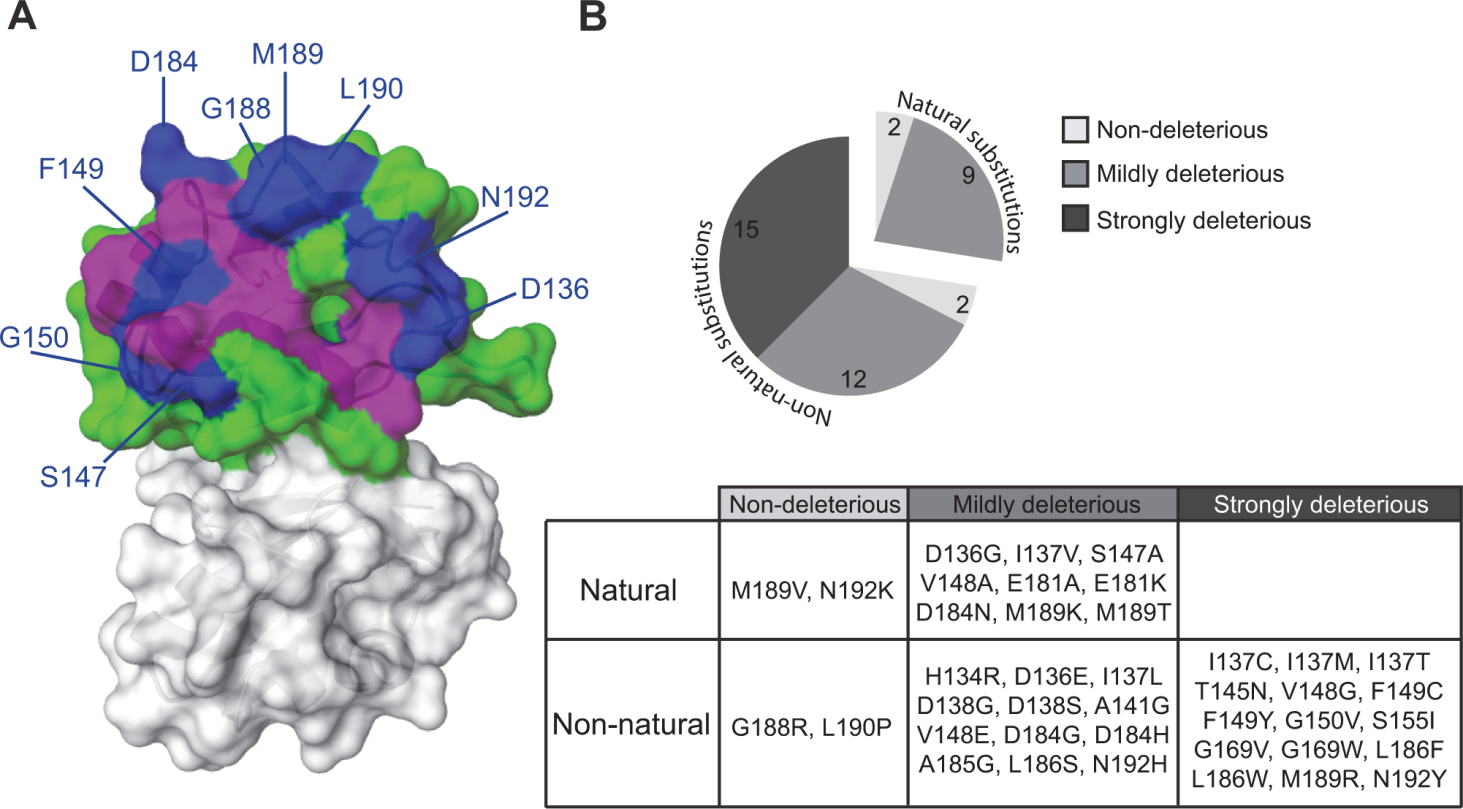
Effects of single amino acid substitutions in the Pab1 RRM2 domain on its interaction with eIF4G1. (A) The crystal structure of the human RRM2 (PDB_ID 1CVJ) is shown. RRM2 positions with at least one amino acid substitution resulting in an enrichment score lower than 50% of the wild-type are colored in purple if the position was discovered by the combined analysis or in blue if the position was identified solely by the two-hybrid assay. All other RRM2 positions are shown in green. (B) Distribution of enrichment scores for amino acid substitutions that were not identified by the combined approach.

While combining natural variation with *in vivo* deep mutational scanning highlights the contribution to protein-protein interactions of residues that change over evolutionary time, it overlooks highly conserved residues and ignores the effects of substitutions to amino acids that do not appear in homologues. We therefore sought to study the effects of mutations on Pab1 RRM2–eIF4G1 association by an alternative approach. To this end, we performed a large-scale two-hybrid analysis. We expressed each of three libraries of RRM2 as a DNA-binding domain hybrid, with mutations covering Pab1 positions 131–150, 151–175 or 176–197, and tested for the binding of these hybrids to the yeast eIF4G1 expressed as an activation domain hybrid. Samples were collected before (input) and after (selected) two-hybrid selection, and the library segments were recovered and sequenced. For each variant, the change in its frequency from input to selected pool (i.e. its enrichment score) was determined as previously described [16]. We were able to extract enrichment scores for 802 single amino acid substitutions across the three library segments, which comprise 60% of all possible substitutions (S1 Table). While mutations that disrupt RRM2 structure caused fortuitous activation of the yeast two-hybrid reporter gene, positions that were shown to be sensitive to natural substitutions when tested individually showed similar sensitivities to mutation in this large-scale assay, suggesting that the enrichment scores for mutations that specifically affect the contact site for eIF4G1 were valid (S2 Fig.). In particular, of the 44 mutations that reduced the enrichment score by more than 50% (log_2_ enrichment score < −1), 22 mutations occur at the eight positions that were found by our natural variation analysis to be involved in eIF4G1 binding (I137, D138, A141, T145, V148, E181, A185 and L186); eight mutations occur at the immediate sequence neighbors of these positions (D136, S147, F149 and D184); and 11 mutations occur at residues that show physical but not immediate sequence proximity to these contact site residues (G150, G188, M189, L190 and N192). Overall, in addition to identifying eIF4G1 contact site residues that were elucidated by the combined approach of the *in vivo* mutational data and the natural variation data, the large-scale two-hybrid results highlighted the contribution of residues at the periphery of this site (Fig. 4A). To understand why mutations at these positions were not discovered using our combined approach, we examined the level of natural variation at these sites. While F149 and G150 are fully conserved, the other residues show some degree of variation in Pab1 homologues. Though some of these natural changes interfered with eIF4G1 binding in the two-hybrid assay, none of them showed a strongly deleterious effect *in vivo* (Fig. 4B), suggesting that the central residues of the eIF4G1 binding site are more sensitive to natural variation substitutions *in vivo* than the peripheral ones.

### Evolutionary paths of deleterious substitutions

**Figure 5:**
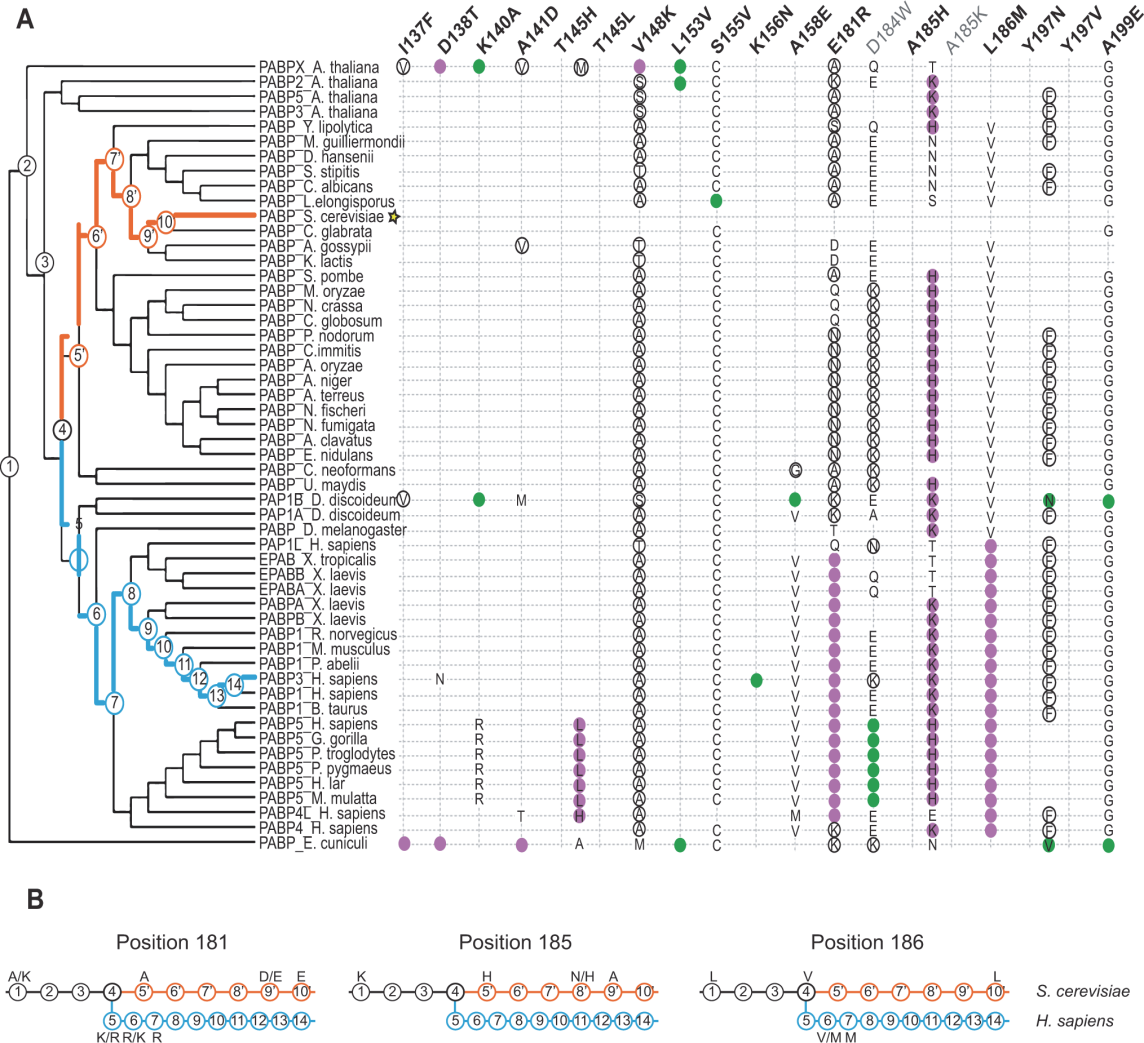
Distribution of single amino acid substitution effects across the Pab1 phylogenetic tree. A) A cladogram describing the phylogenetic lineage of 52 Pab1 homologues. The numbered nodes along the tree represent the common ancestors shared between different branches of the tree that lead to the speciation of PABP-1 orthologues in *S. cerevisiae* and *H. sapiens*. The light blue and the orange paths represent the evolutionary lineages of the human and the yeast, respectively, since the fungal and metazoan poly(A)-binding protein divergence. The position of *S. cerevisiae* in the tree is designated by a star. Each column represents one of the 19 single amino acid substitutions from Pab1 RRM2 homologues that were found to be deleterious. Effects by substitution names depicted in gray are less reliable due to lower input read count. The rows are the Pab1 homologues shown in the phylogenetic tree. A filled circle depicts the presence of the specified substitution in a particular sequence. Purple circles depict mutations that interfere with eIF4G binding and black circles represent mutations that had no effect on eIF4G binding. For two deleterious mutations that occur at the same position, the identities are specified within the colored circle. Also specified are the identities of other amino acids found at the same sites as the deleterious mutations that are either mildly deleterious (open black circle) or non-deleterious (all others). The absence of any marking for a given homologue indicates that the same residue is present in *S. cerevisiae* at that position. B) The common ancestors from the *S. cerevisiae* and the *H. sapiens* lineages shown in (A) are displayed. For each of the designated positions the most probable amino acid is shown (see Material and Methods for reconstruction probability cutoff). Two amino acids are shown with the most likely amino acid designated on the right, if both have high probability score but neither is above the reconstruction probability cutoff. Ancestral sequences with no designated amino acid share the same amino acid identity with the former ancestral sequence.

To understand how incompatible Pab1 variants have evolved in different lineages, we constructed a maximum likelihood tree from the 52 Pab1 homologues. In agreement with theoretical expectations [28], we found that the number of substitutions in Pab1 that were strongly deleterious in *S. cerevisiae* increases with evolutionary distance (Fig. 5A). Specifically, while closely related fungi provide zero or one strongly deleterious substitution, the microsporidian *Encephalitozoon cuniculi*, which carries the most diverse PABP sequence, contributes six deleterious substitutions. The deep divergence of *E. cuniculi* PABP, likely due to rapid evolution of microsporidia after branching off the fungal lineage [29], provides a unique set of mutations (I137F, D138T and A141D) that interfered with eIF4G1 binding. However, unlike the metazoan substitutions that interfered with this binding, the *E. cuniculi* substitutions localize to helix α1 (Fig. 3B), which suggests two alternative paths of eIF4G-binding site evolution. In addition, the deleterious effects of substitutions T145L and T145H, from the non-yeast paralogues of the poly(A) binding protein (PABP5 and PABP4L), reveal the critical function of T145 in eIF4G binding. Taken together, these results highlight the need to analyze evolutionarily remote sequences in order to obtain a detailed map of functional sites in proteins.

The functional scores of the natural substitutions that occurred throughout evolution suggest ancestral states that were likely to promote the divergence of the eIF4G1-binding site. In particular, for position 185, we observe a stepwise decrease in charge in the *S. cerevisiae* lineage, from lysine through histidine and asparagine to alanine (Fig. 5B, middle). Both A185K and A185H were strongly deleterious in yeast, suggesting that the lack of positive charge in yeast was accompanied by other changes in eIF4G or in Pab1 orthologues that are no longer compatible with the ancestral state of this position. At positions 181 and 186, substitutions matching variation within the *S. cerevisiae* lineage were mildly deleterious or non-deleterious, while substitutions matching variation that occurred after the fungal–metazoan divergence were strongly deleterious. Therefore, changes in eIF4G or in Pab1 orthologues that compensate for the otherwise detrimental effects of these mutations are likely to be conserved along the metazoan branch of the tree.

### Changing Pab1 RRM2 binding specificity from yeast eIF4G1 to human eIF4G1

**Figure 6:**
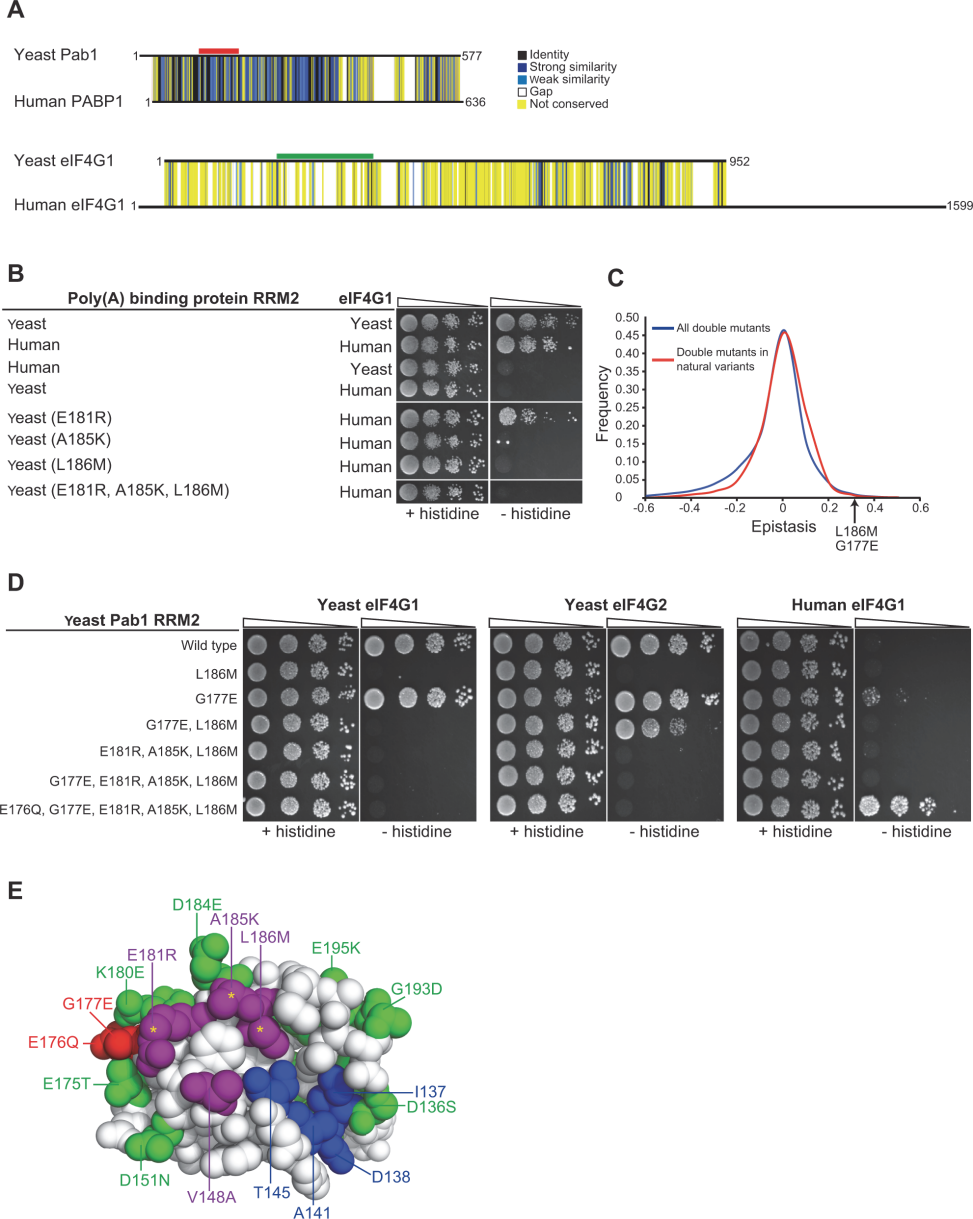
Testing humanizing substitutions for their ability to change the binding specificity of the yeast Pab1 RRM2 to the human eIF4G1. A) Pairwise alignments of the yeast and the human orthologues of the poly(A)-binding protein-1 and eIF4G1 were generated using Clustal Omega [43]. Black horizontal lines designate the protein sequences. Degrees of similarity between aligned positions as well as gaps are color coded with the color key depicted on the left. The yeast Pab1 sequence that is associated with eIF4G1 binding [22] is highlighted with a red line and the yeast eIF4G1 region associated with Pab1 binding [38] is shown with green. B) Yeast two-hybrid assays testing Pab1 RRM2 variants that carry the specified mutations for their ability to interact with the N-terminus (amino acids 1–260) of the human eIF4G1. Cells were serially diluted and seeded as described in Fig. 2. C) Epistasis score distributions of Pab1 variants carrying two mutations that are either found together in natural homologues of the poly(A)-binding protein (red line) or not (blue line). A positive epistasis score indicates a growth rate of the double mutant that was better than expected based on the effects of each of the constituent single mutants alone. An arrow shows the epistasis score of the G177E and L186M double mutant. D) Yeast two-hybrid assays testing Pab1 RRM2 variants that carry the specified mutations for their ability to interact with the N-termini of the yeast eIF4G1 and eIF4G1 and with the human eIF4G1. E) An upper view of the eIF4G1 contact surface of the human PABP1 RRM2 domain (PDB ID 1CVJ). Differences between the yeast and the human sequences are specified by the amino acid substitution nomenclature. eIF4G1 contact site residues that are conserved between yeast and human are shown in blue and contact site residues that differ between the two species are in purple. Deleterious substitutions are marked with yellow asterisks and non-deleterious substitutions are colored in green. E176Q and G177E are shown in red. All other conserved residues between yeast and human are white.

We asked whether the yeast Pab1 and eIF4G protein sequences might enable us to infer the compensatory changes that allowed the establishment of the strongly deleterious substitutions E181R, A185K and L186M in the human orthologue of Pab1. For instance, a pair of mutations comprising one humanizing substitution in yeast Pab1 that interferes with yeast eIF4G1 association and a compensating, second humanizing mutation in the yeast eIF4G1 might restore binding. However, the identification of candidate humanizing substitutions in the yeast eIF4G1 that may form deleterious–compensatory clusters with humanizing mutations in Pab1 is challenging due to the extreme diversification of eIF4G1 and its contact site residues throughout evolution (Fig. 6A). Thus, we decided to explore the inter-protein interactions in Pab1 that underpin the binding of the RRM2 domain to either the yeast or human eIF4G1. While the human and yeast RRM2 domains interacted with their cognate eIF4G1 fragment, neither bound to its non-cognate eIF4G1 fragment (Fig. 6B), suggesting that eIF4G1 binding specificity is dependent on the 25 positions that differ between the yeast and the human RRM2 domains.

We tested a few humanizing mutations in Pab1 RRM2 for their ability to change the binding specificity towards human eIF4G1. Though there are many possible combinations of humanizing substitutions, we used the deep mutational scanning results to narrow down the list of candidate residues. We first evaluated the ability of Pab1 RRM2 fragments that carry each of the three humanizing substitutions (E181R, A185K and L186M) that abolished binding to the yeast eIF4G1 to bind the human eIF4G1 fragment. The E181R variant activated the two-hybrid reporter gene (Fig. 6B), indicating that despite other sequence differences, elements within the yeast Pab1 RRM2 domain support this change in binding specificity. Unlike E181R, A185K and L186M did not bind to human eIF4G1, suggesting that these two substitutions require other humanizing changes in Pab1 RRM2 to function. Combining A185K and L186M with E181R to form a triple mutant did not enable binding of yeast Pab1 to human eIF4G1 (Fig. 6B). Because this triple mutant carries all of the strongly deleterious substitutions that differ between the human and the yeast Pab1 RRM2 domain, this finding suggests that some of the remaining mildly deleterious or non-deleterious substitutions are necessary to compensate for the detrimental effects of A185K and L186M on eIF4G1 binding.

Because the deep mutational scanning of Pab1 RRM2 provided functional scores for multiple variants that change two amino acids [16], we realized that the contribution of other humanizing substitutions to the function of contact site residues might be inferred from the epistasis scores of such variants. We calculated epistasis by taking the enrichment score of a double mutant and subtracting the product of the scores of the component single mutants. Humanizing substitutions that compensate for the deleterious effects of E181R, A185K or L186M are likely to show positive epistasis (*i.e.* the double variant functions better than predicted) while humanizing substitutions that do not should display no epistasis. We extracted the epistasis scores for 866 double mutants ([16], S2 Table), each carrying two substitutions that are found in one of the 52 homologues of Pab1. Comparing the epistasis score distribution of these variants to that of 38,742 double mutants that carry pairs of mutations that do not occur in any of the individual homologues of Pab1 that were sampled in our analysis revealed a small yet significant increase (Wilcoxon rank sum test p-value = 3.712e-10) in epistatic interactions between substitutions that are present together in natural variants (Fig. 6C). Thus, two mutations found in a natural protein variant are more likely to interact positively, either by synergistic or compensatory mechanisms.

Of the 866 double mutants with two substitutions found in Pab1 homologues, eight carry one of the strongly deleterious humanizing substitutions together with a second humanizing mutation (S2 Table). Of these, a double mutant carrying the deleterious substitution L186M together with the non-deleterious substitution G177E had a high epistasis score (Fig. 6C). Specifically, while L186M alone resulted in ∼30% loss of *in vivo* function, addition of the non-deleterious G177E substitution restored Pab1 function to the wild-type level (S2 Table). G177E was able to partly restore eIF4G2 binding of an RRM2 mutant that carries the L186M substitution (Fig. 6D), suggesting that the positive epistasis of G177E and L186M is at least in part due to an improved association of the double mutant with eIF4G2. While adding G177E to the triple mutant did not shift the binding specificity towards the human eIF4G1, humanizing its adjacent residue by E176Q substitution supported this switch (Fig. 6D), suggesting that the local humanized environment of G177E is important for its function. The contribution of E176Q and G177E to human eIF4G1 binding is specific, as other groups of humanizing substitutions, found either at a distance or in close physical proximity to the three deleterious substitutions, were not able to promote this shift in binding specificity (S3A Fig.). Thus, despite the lack of measurable effects of single amino acid substitutions at position 177 of yeast Pab1 (Fig. 1A), the amino acid at this position is important for Pab1 binding to the human eIF4G1. The ancestral state of position 177 in the Pab1 lineage was glutamic acid, which was replaced by glycine in the recent ancestor of *S. cerevisiae* (S3B Fig.). Therefore, it is likely that the pre-establishment of glutamic acid at position 177 compensated in human for the detrimental effects of at least one of the three deleterious substitutions, while becoming dispensable in the evolutionary path that was taken by *S. cerevisiae*.

### Deleterious mutations that map to other functional sites

Of the other nine natural and strongly deleterious substitutions in Pab1, five (K140A, L153V, S155V, K156N and A158E) map to the interface between RRM1 and RRM2. In particular, L153 and K156, present in the human orthologue, are key residues in the interaction between RRM1 and RRM2 that allow for efficient poly(A) binding [30]. In addition, an allosteric change in the RRM1 and RRM2 interface upon poly(A) binding is suggested to facilitate the association of RRM2 with eIF4G [31]. Therefore, deleterious substitutions in the RRM1–RRM2 contact site are likely to result from loss of either poly(A) or eIF4G binding activity, or both.

Three other substitutions (Y197N, Y197V and A199E) map to the poly(A)-binding site [30]. Residue 197 is the only RNA-binding residue that is highly divergent, as all the other residues that bind RNA are either identical across the 52 homologues or display a small variation that is highly tolerated by the yeast protein. It is likely that the structure of the poly(A) forces extreme conservation on the RNA-binding residues, similar to enzyme-substrate binding sites [32], in a way that prevents useful characterization by natural substitutions.

## Discussion

We used the deep mutational scanning data on the yeast Pab1 RRM2 domain to delineate the functional consequences of 197 single amino acid substitutions to residues that occur in Pab1 homologues. As expected [33,34], the great majority of these natural substitutions had a minor effect on Pab1 activity, indicating that the primary constraints on poly(A)-binding protein function remain the same among different organisms. Of the 17 strongly deleterious substitutions, nearly all mapped to either protein-protein (RRM2–eIF4G), inter-domain (RRM2–RRM1) or protein-RNA (RRM2–poly(A)) interaction sites, suggesting that all known ligand-binding sites in Pab1 RRM2 experienced some degree of divergence over evolutionary time.

We characterized the eIF4G-binding site in Pab1 at single amino acid resolution, demonstrating that integrating results from mutagenesis with natural variation data provides a compact list of mutations that are likely to interfere with protein-ligand interaction. The rapid generation of this list overcomes limitations of other mutagenesis approaches. In particular, deletion experiments are unlikely to provide an accurate map of the eIF4G contact site in Pab1, as its critical residues span most of the primary sequence of RRM2 and are brought together by the three-dimensional structure. An alanine scan, which tests the effects of substituting single amino acids to alanine, would prove insufficient to identify the involvement of T145, V148 and E181 in eIF4G binding, as judged by the minor effects of these alanine changes on the *in vivo* function of Pab1 [16].

Although our combined approach delineated eight Pab1 RRM2 surface residues that are associated with eIF4G1 binding, the large-scale two-hybrid assay identified nine additional residues, located mostly at the periphery of the contact site. The higher sensitivity of the RRM2 domain to mutations at the eIF4G1 contact site in the two-hybrid assay is likely due to the higher selection pressure in this assay, than *in vivo*, for the RRM2 eIF4G1 interaction, as mutations that disrupt this interaction are not lethal [12,16,22]. Nonetheless, the differences between the regions that were highlighted by the two approaches point to the central residues, discovered by our combined approach, as more important to the *in vivo* function of the eIF4G1 binding site than the peripheral residues, added by the yeast two-hybrid assay. This difference in the importance of residues to the interaction is likely to mirror the higher evolutionary conservation of the central binding site residues compared to the peripheral ones [35].

Despite the greater ability of the two-hybrid assay to uncover positions on the RRM2 surface that associate with eIF4G1 binding, our combined approach of using natural variation to filter the deep mutational scanning results on the *in vivo* function of RRM2 yields an increased fraction of mutations that interfere with eIF4G1 binding, Because large-scale mutational data are usually not available for a protein interaction, these results emphasize the advantage of this combined approach to identify the effects of mutations on protein interactions. In addition, while mutations that damage the structure of a protein can affect the two-hybrid readout, the short list of candidate mutations created by the *in vivo* approach is likely to exclude such indirect effects [8]

### Implications for Pab1 and eIF4G1 activity

Elucidating contact sites with high resolution is important to clarify how proteins exert their functions. With respect to Pab1, we found that the binding sites for eIF4G1 and eIF4G2 extend beyond the helix α2 element [12] to include part of helix α1. The inclusion of this helix provides a plausible explanation for the molecular mechanism that couples poly(A) and eIF4G binding by Pab1. In yeast, binding of eIF4G to Pab1 requires the prior association of Pab1 with poly(A) in order to promote translation [22]. In human, these sequential steps are separated by inter-domain allostery of RRM2 and RRM1, allowing PABP1 to adopt a more extended conformation in the presence of RNA [31]. Since the association of RRM2 and RRM1 involves direct interactions between helix α1 of RRM2 and helix α2 of RRM1 [30,31], conformational changes of the two domains might make helix α1 of RRM2 and its surrounding residues available for eIF4G association upon poly(A) binding. Our finding that a Pab1 fragment consisting only of RRM1–RRM2 was unable to bind eIF4G supports the regulatory role of this inter-domain interaction in this function.

eIF4G1 and eIF4G2 are functionally interchangeable under optimal growth conditions [36]. However, differences in eIF4E co-purification and *in vitro* translation efficiencies suggest that each of the two isoforms possesses unique roles in translation under non-optimal conditions [37,38]. Despite the overlap in location and similar mutational sensitivity of the binding sites for eIF4G1 and eIF4G2, a few Pab1 RRM2 substitutions resulted in differential sensitivities to binding. Whether this difference in Pab1 RRM2 binding points to altered mechanisms of action is a matter of further studies. T145L, which bound only to eIF4G2, might be useful in clarifying specific roles for the isoforms in translation.

### Implications for the evolution of binding sites

We identified three substitutions (E181R, A185K and L186M), corresponding to the residues present in the human PABP1, each of which when introduced into the yeast Pab1 eliminated its binding to yeast eIF4G1. We tested whether these substitutions might switch the specificity of Pab1 to bind human eIF4G1. The single humanizing substitution, E181R, allowed the yeast Pab1 RRM2 to bind to human eIF4G1, demonstrating that in spite of sequence diversification, the human and yeast orthologues of eIF4G1 and Pab1 share similarities with respect to their physical association. However, Pab1 carrying A185K and L186M did not bind to the human eIF4G1, even after humanizing the contact site by other substitutions. Thus, this shared similarity in binding activity is likely to be maintained by other intra-protein interactions in Pab1 that compensate for the otherwise deleterious effect of A185K and L186M.

Our finding that two substitutions that are both present in an individual homologue are more likely to display positive epistatic interactions suggests that compensating mutations reconstruct functional modules that are conserved between organisms despite changes in the amino acid sequence that comprise these modules. Indeed, that the addition of the G177E substitution repairs the binding of an RRM2 L186M mutant to the yeast eIF4G2 suggests that the two humanizing substitutions restore a functional binding site for the yeast eIF4G2. Additional studies will be required to determine whether the tendency for positive epistasis of two substitutions present in a homologous sequence is a universal property of proteins or a specific feature of Pab1. Nonetheless, it is likely that substitutions from paralogues of closely related species are more prone to this type of epistasis than substitutions from other homologous sequences, given the functional conservation and the small number of amino acid changes in these paralogues.

Additionally, G177E together with E176Q combined with the three deleterious substitutions E181R, A185K and L186M to allow yeast Pab1 binding to the human eIF4G1. This finding supports the use of epistatic interactions between two natural substitutions tested in a model organism to infer similar interactions between those residues in their natural context. We suggest that systematic integration of large-scale epistasis data with bioinformatic tools that use sequence homology might improve prediction accuracies of co-evolutionary relationships and functional association between residues.

### Conclusions

Approximately 20% of *S. cerevisiae* genes are essential for growth on rich glucose medium [39], with many of the remaining genes required upon environmental or genetic perturbations. Therefore, growth selections compatible with deep mutational scanning can be used to study the *in vivo* function of a large fraction of yeast proteins. This experimental strategy can also be applied to cross-species complementation assays to analyze human proteins in yeast [9,40,41]. However, the score assigned to each protein variant reflects the consequence of mutation only on growth rate. Therefore, inferring the direct impact of mutations on an *in vivo* activity such as ligand binding remains challenging. Here we show that integrating deep mutational scanning results with natural variation data provides a high throughput inter-species complementation assay that can be used to identify and characterize functional regions in proteins, including protein-protein contact sites. In addition, the large-scale analysis of natural amino acid substitutions can provide an experimental platform to evaluate the performance of computational tools that use protein homology to predict function and co-evolutionary relationships.

## Materials and Methods

### Deep mutational scanning

The procedures for Pab1 RRM2 deep mutational scanning, including establishment of the experimental platform, construction of mutant libraries, sequencing of RRM2 DNA fragments and data analysis were previously described [16]. Unless otherwise indicated, only variants with input-read counts greater than 40 were used for the analysis.

### Plasmids

pOBD2 and pOAD were used to test the interactions between Pab1 and eIF4G isoforms in the yeast two-hybrid system. Full length *PAB1* encoding amino acids 1–578 (DMP87) was PCR amplified from pCM188-Pab1 [16] and cloned into the NcoI and SalI sites of pOBD2. The following *PAB1* truncations, encoding amino acids 1–343 (DMP88), 1–204 (DMP183), 123–204 (DMP180) and 1–120 (DMP179) were PCR amplified from p415GPD-Pab1-343BX [16] and cloned into the NcoI and SalI sites of pOBD2. *PAB1* fragments encoding amino acids 123–204 (RRM2) with the point mutations I137F (DMP201), D138T (DMP202), K140A (DMP203), A141D (DMP230), T145H (DMP204), T145L (DMP189), V148K (DMP205), L153V (DMP206), S155V (DMP207), K156N (DMP208), A158E (DMP209), K180E (DMP197), E181R (DMP193), D184E (DMP188), D184W (DMP210), A185H (DMP211), A185K (DMP186), L186M (DMP185), Y197N (DMP191), Y197V (DMP194), A199E (DMP192), [K180E, E181R] (DMP198), [D184E, A185K, L186M] (DMP190), [E181R, A185K, L186M] (DMP235), [E181R, A185K, L186M, A158V, T159C] (DMP286), [E181R, A185K, L186M, E176Q, G177E] (DMP287), [E181R, A185K, L186M, V148A, K180E, D184E] (DMP291), [E181R, A185K, L186M, P135K, Q194R] (DMP292), G177E (DMP293), [G177E, L186M] (DMP297) and [E181R, A185K, L186M, G177E] (DMP298) were created by PCR using the same p415GPD-Pab1-343BX plasmid as a template and cloned into the NcoI and SalI sites of pOBD2, C-terminal and in-frame with the Gal4 DNA binding domain. eIF4G1 and eIF4G2 fragments encoding amino acids 1–341 were amplified from yeast genomic DNA (strain W-303) and cloned into the EcoRI and SalI sites of pOAD, C-terminal and in-frame with the Gal4 activation domain (DMP92 and DMP212, respectively). The human PABP1 fragment encoding amino acids 95–176 was amplified from HsCD00042197 (PlasmidID) and cloned into the NcoI and SalI sites of pOBD2 (DMP264). The human eIF4G1 fragment encoding amino acids 1–260 was amplified from HsCD00342900 (PlasmidID) and cloned into the NcoI and SalI sites of pOAD (DMP265)

### Individual yeast two-hybrid assays

Yeast strain PJ694a (*MATa trp1-901 leu2-3,112 ura3-52 his3-200 gal4Δ gal80Δ LYS2::GAL1-HIS3 GAL2-ADE2 met2::GAL7-lacZ*) carrying pOBD2- and pOAD-based vectors were grown overnight, at 30°C in synthetic complete media lacking leucine and tryptophan. To test for activation of the *HIS3* reporter gene, cells were spotted in a dilution series on synthetic complete plates lacking leucine and tryptophan, with or without histidine and grown at 30°C for three days.

### Data for natural single amino acid variations

We collected 52 Pab1 homologues (see S1 File for sequences and accession numbers), representing sequences of all poly(A)-binding proteins that carry four consecutive RRM domains that can be found in the UniProtKB/SwissProt database [42], which contains high quality annotations of protein sequences. Multiple sequence alignment (MSA) was performed using Clustal Omega [43] with default parameters (S2 File). Enrichment scores for natural and non-natural single amino acid substitutions were obtained from Supplementary Table 2 of Melamed et al [16].

### Properties of natural and non-natural amino acid substitutions

To assess the fraction of natural substitutions that result in impaired function, enrichment score distributions of 160 natural single amino acid substitutions, 539 non-natural single amino acid substitutions and 229 synonymous variants with input read counts greater than 500 were determined. The stringent input read count threshold was set to minimize fluctuations of enrichment scores due to low representation of variants in the library pools. The enrichment scores distribution of the synonymous variants was used as a proxy for the enrichment scores distribution of non-deleterious variants in the dataset. To estimate the fraction of deleterious substitutions within the natural substitutions, for each enrichment score bin shown in S1B Fig., we subtracted the estimated fraction of non-deleterious variants from the fraction of the natural variants.

For each single amino acid substitution, the fractional accessible surface area (ASA) was obtained for the side chains of the wild-type residue in the human PABP1 RRM2 structure (PDB ID 2K8G) using VADAR server, version 1.8 [44]. Data for K164 residue was omitted, as this residue is absent from the human RRM2 (see Fig. 2A). The Blosum62 matrix was used to score each substitution to determine the degree of conservation. Box plots were generated using R-studio software.

### Large-scale yeast two-hybrid assays

RRM2 sequences containing one of the three library segments were PCR amplified from the library plasmids that were previously described [16] and cloned into the NcoI and SalI sites of pOBD2. Yeast expressing the RRM2 hybrid containing one of the three libraries were grown to log phase in SC medium lacking leucine and tryptophan, supplemented with 2% glucose, and diluted into fresh medium lacking leucine, tryptophan and histidine to a final concentration of 4 × 10^4^ cells/mL. Selection was carried out for 21 h with the culture growing to a density of 5 × 10^6^–1 × 10^7^ cells/mL. 2.5 × 10^8^ cells from each culture were collected before (“input”) and after selection (“selected”). Library preparation for high throughput sequencing was carried as previously described [16]. Amplicons were created with internal primers that flanked each library segment and carried at their 5’ end common sequencing targets for Illumina read1, read2 and index primers (11 PCR cycles) and with external primers that added Illumina adapter sequences (8 PCR cycles). Amplicons were sequenced by an Illumina NextSeq500 using paired-end reads. We used the Enrich software package (Fowler et al. 2011) to filter for high quality reads (base Q score >20). Based on the variance of enrichment scores of 2423 synonymous variants (i.e. variants that encode the wild-type Pab1 RRM2 protein sequence and carry at least one synonymous mutation), we selected variants with at least 20 input read counts for further analysis (synonymous variance <0.4 for all three libraries). Enrichment scores of single amino acid substitutions were log2 transformed and visualized using Java TreeView 1.1.6r2 [45]. Average linkage hierarchical clustering with a Euclidean distance similarity metric for both RRM2 residues and substituting amino acids was performed using Gene Cluster 3.0 [46].

### Construction of a phylogenetic tree and determination of ancestral states

A maximum likelihood tree was constructed using the Phylogeny.fr tool [47] using default parameters. Probabilities for ancestral states were calculated using the JTT model of substitution by the FastML tool [48]. Ancestral amino acids were considered “true” if their reconstruction probabilities were greater than 0.7 (the sum of probabilities for all amino acids equals 1.0). Otherwise, the two most probable amino acids with a minimal probability of 0.3 for each, and sum of probabilities greater than 0.75 were considered.

### Structure visualization

The human PABP1 RRM1-RRM2 structure (PDB ID 1CVJ) was visualized using PyMol software (version 1.5.0.5).

## Supporting Information

S1 Fig Enrichment scores distribution of synonymous mutations to assess the contamination of the mildly and strongly deleterious groups by non-deleterious mutants. A) A bar graph shows the enrichment score distributions of natural (orange), non-natural (white), and synonymous variants (gray) with input read counts greater than 500. B) Natural and synonymous variants falling into the non-deleterious, mildly deleterious and strongly deleterious categories were counted (Synonymous Observed and Natural Observed rows). The expected number of natural variants in each category based on the distribution of synonymous variants, which serve as a proxy for non-deleterious variants, is shown (Natural Expected). For the natural substitution variants, the ratio between the expected and the observed numbers in each category represents the estimated fraction of contamination by non-deleterious mutants.
(EPS)

S2 Fig Effects of single amino acid substitutions in the RRM2 domain on the two-hybrid interaction for RRM2–eIF4G1 binding. A) A heat map showing the enrichment scores (log_2_ transformed) for single amino acid substitutions in the Pab1 RRM2 sequence. Each column represents a site in the RRM2 sequence and each row a substitution to a specific amino acid. An asterisk designates nonsense mutations. Color ranges from blue for the most detrimental mutations to red for the most beneficial. Effects of stop codons are highly detrimental through most of the first library segment, but become beneficial if they occur C-terminal to helix-α1. In addition, substitutions to proline, which were the most deleterious *in vivo* due to their presumed interference with α-helix and β-sheet structures [16], were highly enriched in this assay. B) Clustering the effects of single amino acid substitutions based on enrichment scores. The yellow frame outlines a cluster of positions that displayed a beneficial effect in response to most substitutions. Below, the locations of these positions in the protein’s core are highlighted in red spheres in the RRM2 structure (PDB_ID 2K8G). All other positions are shown in green. While these observations point to fortuitous activation of two-hybrid transcription due to mutations that disrupt the RRM2 structure, the assay was highly sensitive to mutations in other positions known to be involved in eIF4G1 binding.
(EPS)

S3 Fig A test of humanizing substitutions for their ability to change the binding specificity of the yeast Pab1 RRM2 to the human eIF4G1. A) Yeast two-hybrid assays testing Pab1 RRM2 variants that carry the specified mutations for their ability to interact with the N-terminus (amino acids 1–260) of the human eIF4G1. Cells were serially diluted and seeded as described in Fig. 2. B) The common ancestors of positions 176 and 177 from the *S. cerevisiae* and the *H. sapiens* lineages shown in Fig. 4A are displayed. For each of the designated positions the most probable amino acid is shown (see Materials and Methods for reconstruction probability cutoff).
(EPS)

S1 Table Enrichment scores for single amino acid substitutions in Pab1 RRM2 from mutational analysis of Pab1 RRM2 – eIF4G1 interaction in the yeast two-hybrid system. Enrichment scores are normalized to the wild-type score and expressed as a log_2_ ratio. The RRM2 positions are specified in the most right column and the substituting amino acids are designated on the top row by a single letter nomenclature. Substitutions to stop codons are depicted by an asterisk. Blank cells represent substitutions that did not pass Enrich quality filtration or were identical to wild-type residues.
(TXT)

S2 Table Epistasis scores for double mutants that occur in Pab1 homologues. Epistasis scores for double mutants carrying two mutations that are found in at least one individual homologue of Pab1. Data were extracted from Supplementary Table 5 of Melamed et al [16].
(TXT)

S1 File Pab1 homologous sequences. Sequences of the 52 Pab1 homologues used in this study are shown in Fasta format with UniprotKB headers.
(RTF)

S2 File Multiple sequence alignment (MSA) of Pab1 homologues. MSA of Pab1 homologues shown in Clustal format. Strongly deleterious substitutions are displayed in lowercase.
(TXT)
