# Supplementary material for: Combining Natural Sequence Variation with High Throughput Mutational Data to Reveal Protein Interaction Sites
Source: PLoS Genet. 2015 Feb 11;11(2):e1004918. doi: 10.1371/journal.pgen.1004918 (PMC4335499; doi:10.1371/journal.pgen.1004918)
Supplement: S1 File — Pab1 homologous sequences. Sequences of the 52 Pab1 homologues used in this study are shown in Fasta format with UniprotKB headers. (RTF) [file pgen.1004918.s006.rtf]

>sp|Q96DU9|PABP5_HUMAN Polyadenylate-binding protein 5 OS=Homo sapiens GN=PABPC5 PE=1 SV=1MGSGEPNPAGKKKKYLKAALYVGDLDPDVTEDMLYKKFRPAGPLRFTRICRDPVTRSPLGYGYVNFRFPADAEWALNTMNFDLINGKPFRLMWSQPDDRLRKSGVGNIFIKNLDKSIDNRALFYLFSAFGNILSCKVVCDDNGSKGYAYVHFDSLAAANRAIWHMNGVRLNNRQVYVGRFKFPEERAAEVRTRDRATFTNVFVKNIGDDIDDEKLKELFCEYGPTESVKVIRDASGKSKGFGFVRYETHEAAQKAVLDLHGKSIDGKVLYVGRAQKKIERLAELRRRFERLRLKEKSRPPGVPIYIKNLDETINDEKLKEEFSSFGSISRAKVMMEVGQGKGFGVVCFSSFEEATKAVDEMNGRIVGSKPLHVTLGQARRRC>sp|Q05196|PABP5_ARATH Polyadenylate-binding protein 5 OS=Arabidopsis thaliana GN=PAB5 PE=1 SV=3MAAAVASGIAPTTAMVDQVIPNQPTVAAAAPPPFPAVSQVAAVAAAAAAAEALQTHPNSSLYVGDLDPSVNESHLLDLFNQVAPVHNLRVCRDLTHRSLGYAYVNFANPEDASRAMESLNYAPIRDRPIRIMLSNRDPSTRLSGKGNVFIKNLDASIDNKALYETFSSFGTILSCKVAMDVVGRSKGYGFVQFEKEETAQAAIDKLNGMLLNDKQVFVGHFVRRQDRARSESGAVPSFTNVYVKNLPKEITDDELKKTFGKYGDISSAVVMKDQSGNSRSFGFVNFVSPEAAAVAVEKMNGISLGEDVLYVGRAQKKSDREEELRRKFEQERISRFEKLQGSNLYLKNLDDSVNDEKLKEMFSEYGNVTSCKVMMNSQGLSRGFGFVAYSNPEEALLAMKEMNGKMIGRKPLYVALAQRKEERQAHLQSLFTQIRSPGTMSPVPSPMSGFHHHPPGGPMSGPHHPMFIGHNGQGLVPSQPMGYGYQVQFMPGMRPGAGPPNFMMPFPLQRQTQPGPRVGFRRGANNMQQQFQQQQMLQQNASRFMGGAGNRRNGMEASAPQGIIPLPLNASANSHNAPQRSHKPTPLTISKLASDLALASPDKHPRMLGDHLYPLVEQQEPANAAKVTGMLLEMDQAEILHLLESPEALKAKVSEALDVLRRSADPAAVSSVDDQFALSSSE>sp|P42731|PABP2_ARATH Polyadenylate-binding protein 2 OS=Arabidopsis thaliana GN=PAB2 PE=1 SV=1MAQVQLQGQTPNGSTAAVTSAPATSGGATATQFGNTSLYVGDLDFNVTDSQLFDAFGQMGTVVTVRVCRDLVTRRSLGYGYVNFTNPQDAARAIQELNYIPLYGKPIRVMYSHRDPSVRRSGAGNIFIKNLDESIDHKALHDTFSSFGNIVSCKVAVDSSGQSKGYGFVQYANEESAQKAIEKLNGMLLNDKQVYVGPFLRRQERDSTANKTKFTNVYVKNLAESTTDDDLKNAFGEYGKITSAVVMKDGEGKSKGFGFVNFENADDAARAVESLNGHKFDDKEWYVGRAQKKSERETELRVRYEQNLKEAADKFQSSNLYVKNLDPSISDEKLKEIFSPFGTVTSSKVMRDPNGTSKGSGFVAFATPEEATEAMSQLSGKMIESKPLYVAIAQRKEDRRVRLQAQFSQVRPVAMQPSVGPRMPVYPPGGPGIGQQMFYGQAPPAMIPPQPGYGYQQQLVPGMRPGGGPVPSFFMPMVQPQQQRPGGGRRPGGIQHSQQQNPMMQQQMHPRGRMFRYPQGRGGSGDVPPYDMGNNMPLTIGALASNLSNATPEQQRTMLGEVLYPLVEQVEAESAAKVTGMLLEMDQTEVLHLLESPEALKAKVAEAMDVLRSVAAGGATEQLASLNLS>sp|O64380|PABP3_ARATH Polyadenylate-binding protein 3 OS=Arabidopsis thaliana GN=PAB3 PE=2 SV=1MAAAVATGVAPATMVDQVPSPTAQTSVQVPVSIPLPSPVVVADQTHPNSSLYAGDLDPKVTEAHLFDLFKHVANVVSVRVCRDQNRRSLGYAYINFSNPNDAYRAMEALNYTPLFDRPIRIMLSNRDPSTRLSGKGNIFIKNLDASIDNKALFETFSSFGTILSCKVAMDVTGRSKGYGFVQFEKEESAQAAIDKLNGMLMNDKQVFVGHFIRRQERARDENTPTPRFTNVYVKNLPKEIGEDELRKTFGKFGVISSAVVMRDQSGNSRCFGFVNFECTEAAASAVEKMNGISLGDDVLYVGRAQKKSEREEELRRKFEQERINRFEKSQGANLYLKNLDDSVDDEKLKEMFSEYGNVTSSKVMLNPQGMSRGFGFVAYSNPEEALRALSEMNGKMIGRKPLYIALAQRKEDRRAHLQALFSQIRAPGPMSGFHHPPGGPMPGPPQHMYVGQNGASMVPSQPIGYGFQPQFMPGMRPGSGPGNFIVPYPLQRQPQTGPRMGFRRGATNVQQHIQQQQLMHRNPSPGMRYMNGASNGRNGMDSSVPQGILPPIIPLPIDASSISHQKAPLLPISKLTSSLASASPADRTRMLGEQLYPLVERHEPLHVAKVTGMLLEMDQAEILHLMESPEALKSKVSEALDVLRLSVDPTDHDLGFSTTD>sp|Q9H361|PABP3_HUMAN Polyadenylate-binding protein 3 OS=Homo sapiens GN=PABPC3 PE=1 SV=2MNPSTPSYPTASLYVGDLHPDVTEAMLYEKFSPAGPILSIRICRDLITSGSSNYAYVNFQHTKDAEHALDTMNFDVIKGKPVRIMWSQRDPSLRKSGVGNIFVKNLDKSINNKALYDTVSAFGNILSCNVVCDENGSKGYGFVHFETHEAAERAIKKMNGMLLNGRKVFVGQFKSRKEREAELGARAKEFPNVYIKNFGEDMDDERLKDLFGKFGPALSVKVMTDESGKSKGFGFVSFERHEDAQKAVDEMNGKELNGKQIYVGRAQKKVERQTELKRTFEQMKQDRITRYQVVNLYVKNLDDGIDDERLRKAFSPFGTITSAKVMMEGGRSKGFGFVCFSSPEEATKAVTEMNGRIVATKPLYVALAQRKEERQAYLTNEYMQRMASVRAVPNQRAPPSGYFMTAVPQTQNHAAYYPPSQIARLRPSPRWTAQGARPHPFQNKPSAIRPGAPRVPFSTMRPASSQVPRVMSTQRVANTSTQTVGPRPAAAAAAAATPAVRTVPRYKYAAGVRNPQQHRNAQPQVTMQQLAVHVQGQETLTASRLASAPPQKQKQMLGERLFPLIQAMHPTLAGKITGMLLEIDNSELLYMLESPESLRSKVDEAVAVLQAHQAKEATQKAVNSATGVPTV>sp|P60047|PABP5_GORGO Polyadenylate-binding protein 5 OS=Gorilla gorilla gorilla GN=PABPC5 PE=3 SV=1MGSGEPNPAGKKKKYLKAALYVGDLDPDVTEDMLYKKFRPAGPLRFTRICRDPVTRSPLGYGYVNFRFPADAEWALNTMNFDLINGKPFRLMWSQPDDRLRKSGVGNIFIKNLDKSIDNRALFYLFSAFGNILSCKVVCDDNGSKGYAYVHFDSLAAANRAIWHMNGVRLNNRQVYVGRFKFPEERAAEVRTRDRATFTNVFVKNIGDDIDDEKLKELFCEYGPTESVKVIRDASGKSKGFGFVRYETHEAAQKAVLDLHGKSIDGKVLYVGRAQKKIERLAELRRRFERLRLKEKSRPPGVPIYIKNLDETINDEKLKEEFSSFGSISRAKVMMEVGQGKGFGVVCFSSFEEATKAVDEMNGRIVGSKPLHVTLGQARRRC>sp|Q7JGR2|PABP5_MACMU Polyadenylate-binding protein 5 OS=Macaca mulatta GN=PABPC5 PE=3 SV=1MGSGEPNPAGKKKKYLKAALYVGDLDPDVTEDMLYKKFRPAGPLRFTRICRDPVTRSPLGYGYVNFRFPADAEWALNTMNFDLINGKPFRLMWSQPDDRLRKSGVGNIFIKNLDKSIDNRALFYLFSAFGNILSCKVVCDDNGSKGYAYVHFDSLAAANRAIWHMNGVRLNNRQVYVGRFKFPEERAAEVRTRDRATFTNVFVKNIGDDIDDEKLKELFCEYGPTESVKVIRDASGKSKGFGFVRYETHEAAQKAVLDLHGKSIDGKVLYVGRAQKKIERLAELRRRFERLRLKEKSRPPGVPIYIKNLDETINDEKLKEEFSSFGSISRAKVMMEVGQGKGFGVVCFSSFEEATKAVDEMNGRVVGSKPLHVTLGQARRRC>sp|P60049|PABP5_PANTR Polyadenylate-binding protein 5 OS=Pan troglodytes GN=PABPC5 PE=3 SV=1MGSGEPNPAGKKKKYLKAALYVGDLDPDVTEDMLYKKFRPAGPLRFTRICRDPVTRSPLGYGYVNFRFPADAEWALNTMNFDLINGKPFRLMWSQPDDRLRKSGVGNIFIKNLDKSIDNRALFYLFSAFGNILSCKVVCDDNGSKGYAYVHFDSLAAANRAIWHMNGVRLNNRQVYVGRFKFPEERAAEVRTRDRATFTNVFVKNIGDDIDDEKLKELFCEYGPTESVKVIRDASGKSKGFGFVRYETHEAAQKAVLDLHGKSIDGKVLYVGRAQKKIERLAELRRRFERLRLKEKSRPPGVPIYIKNLDETINDEKLKEEFSSFGSISRAKVMMEVGQGKGFGVVCFSSFEEATKAVDEMNGRIVGSKPLHVTLGQARRRC>sp|P60050|PABP5_PONPY Polyadenylate-binding protein 5 OS=Pongo pygmaeus GN=PABPC5 PE=3 SV=1MGSGEPNPAGKKKKYLKAALYVGDLDPDVTEDMLYKKFRPAGPLRFTRICRDPVTRSPLGYGYVNFRFPADAEWALNTMNFDLINGKPFRLMWSQPDDRLRKSGVGNIFIKNLDKSIDNRALFYLFSAFGNILSCKVVCDDNGSKGYAYVHFDSLAAANRAIWHMNGVRLNNRQVYVGRFKFPEERAAEVRTRDRATFTNVFVKNIGDDIDDEKLKELFCEYGPTESVKVIRDASGKSKGFGFVRYETHEAAQKAVLDLHGKSIDGKVLYVGRAQKKIERLAELRRRFERLRLKEKSRPPGVPIYIKNLDETINDEKLKEEFSSFGSISRAKVMMEVGQGKGFGVVCFSSFEEATKAVDEMNGRIVGSKPLHVTLGQARRRC>sp|P0CB38|PAB4L_HUMAN Polyadenylate-binding protein 4-like OS=Homo sapiens GN=PABPC4L PE=2 SV=1MNVAAKYRMASLYVGDLHADVTEDLLFRKFSTVGPVLSIRICRDQVTRRSLGYAYVNFLQLADAQKALDTMNFDIIKGKSIRLMWSQRDAYLRRSGIGNVFIKNLDKSIDNKTLYEHFSAFGKILSSKVMSDDQGSKGYAFVHFQNQSAADRAIEEMNGKLLKGCKVFVGRFKNRKDREAELRSKASEFTNVYIKNFGGDMDDERLKDVFSKYGKTLSVKVMTDSSGKSKGFGFVSFDSHEAAKKAVEEMNGRDINGQLIFVGRAQKKVERQAELKQMFEQLKRERIRGCQGVKLYIKNLDDTIDDEKLRNEFSSFGSISRVKVMQEEGQSKGFGLICFSSPEDATKAMTEMNGRILGSKPLSIALAQRH>sp|P60048|PABP5_HYLLA Polyadenylate-binding protein 5 OS=Hylobates lar GN=PABPC5 PE=3 SV=1MGSGEPNPAGKKKKYLKAALYVGDLDPDVTEDMLYKKFRPAGPLRFTRICRDPVTRSPLGYGYVNFRFPADAEWALNTMNFDLINGKPFRLMWSQPDDRLRKSGVGNIFIKNLDKSIDNRALFYLFSAFGNILSCKVVCDDNGSKGYAYVHFDSLAAANRAIWHMNGVRLNNRQVYVGRFKFPEERAAEVRTRDRATFTNVFVKNIGDDIDDEKLKELFCEYGPTESVKVIRDASGKSKGFGFVRYETHEAAQKAVLDLHGKSIDGKVLYVGRAQKKIERLAELRRRFERLRLKEKSRPPGVPIYIKNLDETINDEKLKEEFSSFGSISRAKVMMEVGQGKGFGVVCFSSFEEATKAVDEMNGRIVGSKPLHVTLGQARRRC>sp|Q8SR30|PABP_ENCCU Polyadenylate-binding protein, cytoplasmic and nuclear OS=Encephalitozoon cuniculi (strain GB-M1) GN=PAB1 PE=1 SV=1MEIDESKRASSDSLTIYVGELSPKTLDSDLFRVFSNVGKVLSVKLIKRAEPVSSFAFVTFENEEDAERAIREYKHYELHNRQIRVMKKDERPPETGNIFVKNLPEDFTGKDLDDAFSMFGEIVSCKVATTSHGKSKGYGFVQFKEKKAAKKVIKNFSSLDGLLLGGNRIVVELYNPEIKKGESKKTSATFTNCFIKNFPFDASEAELLELLERYGKVTSLFFPVKDNGKPKGFAFANFENHESALNAIKNLHGTFPFGAGRDGTGEAFYIQKGQRKEERAEELRKMFEQMSMQGQSYKKNLYITNIPEGFGCEELGSIFKEFGNITSISVGVDGANSQKQYAYICYSTPEEASIAVERGNEIYLDGNRLQVAYFKNKLERMKEKEFGGGLGYKPGVPYMYNQGVSFASRGFKRERNRGGAAKPYGNELEKLHSLVLAAAPSFKSQWKDFGVGNEVEFANKVIRAFRSRSEEEIKDMIDLNFVLTKNIASAIEDDNSNDQVLL>sp|Q1ZXC2|PAP1B_DICDI Polyadenylate-binding protein 1-B OS=Dictyostelium discoideum GN=pabpc1B PE=3 SV=1MVPTTESHENNMPDNAAITQQQQDATTSSSSSVATQPPQSQIPPQPQYQYQMAPPPQTHHVHPHHVHPHHQQHPGYVPSHHHHHQQHHHHHPHHVGVPNLHHSIHQQQHPGYVPSHHHHQQQHHHHQHQHQHHQHQHHQHQHHQHHQHHQHHHTSPMGAGAGAAGMPILSMSPIGAYQPPHQLTSLYVGDLAADVNEIILNELFSKVGRNAIASIHVCRDSNTLRSLGYAYVNFFNNHDAERALDTLNYTLVHGKPCRIMWSYRDPTKRKTNVGNIFVKNLEKGVDNAMLYDTFSSFGNILSCKVEFEKGISKGYGYVHFETNDSAEKAIEKVNGTLILGKPINVERFVSKVERYKVENKVFFRNADESITIEILQQELSNRFGEIESCILKNDANGKSKGLGLVEFKNQEDAQKILTESGALIISTIDGTTTVSSNGGTIEINGKPITIDRIKSKVERFTEYRKKTTDLSLFINNIDESIDRDLIKEEFAKHGTIIGIKIVQDENARNKGFGFISFSEIQEAQKALDSLNGFTFGSKQIQVSFSNKDNNQINNKLNGNSTKITKNIIQGGASASQYTGYLPINRYQQHLPHQHINPMYTQQPYFPQQQQSSSSSQPSSSQPQPSSPSHLNGNTTTTSPNTRYSKTLNGTTPFKKSNLPQNANGTNNNNNNNNTNINKSNNTTQSNGFRNKRVPNGKPRYNNNNNSSNNNNNNNTTTNVTTTPSSTETTTPKTTITLEFITNATAEEATETLGSEVYNLVLAKYNNNIELAAKIAGMIVDAVPEHKELFEIISNGQIQSKIEEAKSLLDQPDQE>sp|Q2UK72|PABP_ASPOR Polyadenylate-binding protein, cytoplasmic and nuclear OS=Aspergillus oryzae (strain ATCC 42149 / RIB 40) GN=pab1 PE=3 SV=1MSADASTTPAADSNVTSTPETSTTPAAPAPEVTAVESTTAPNASQPHSASLYVGELDPSVTEAMLYELFSSIGQVASIRVCRDAVTRRSLGYAYVNYNNTADGERALEDLNYTLIKGKPCRIMWSQRDPALRKTGQGNVFIKNLDSAIDNKALHDTFAAFGNILSCKVAQDEFGNSKGYGFVHYETAEAANNAIKHVNGMLLNDKKVFVGHHISKKDRQSKFEEMKANFTNVYIKNIDQDVTEEEFRELFEKFGEITSATLSRDQEGKSRGFGFVNFSTHESAQAAVDEMNEKEIRTQKLYVGRAQKKHEREEELRKQYEAARLEKASKYQGVNLYVKNLTDDVDDEKLRELFGPYGTITSAKVMRDTNIERTQTPESDKEKENKEATKENEKESSEAEKAEKTEEKPADSGDEKKEDKESKKADKKGLGKSKGFGFVCFSSPDEASKAVTEMNQRMVNGKPLYVALAQRKDVRRSQLEASIQARNTIRQQQAAAAAGMPQPYMQPAVFYGPGQQGFIPGQRGGIAFPPQPGMVMAGIPGGRPGQYPGPFPGQQGGRGMGPNQQLPPNFQGIPMGAMQGPVPNGMGYPQGMAQVQFGRGAGGRGQVPGMPNMGQGMRGPGYGQGRGGVPVQQGQMRPGQGGRGQNAAQAPAGRPEEAVAGGLTAQALSAAPPPQQKQMLGEALYPKIQAQQPELAGKITGMLLEMENTELLSLLEDEEALRAKVDEALNVYDEYMKNKGGESEATGEAAKPKEAAKETSTEENKS>sp|Q9ZQA8|PABPX_ARATH Probable polyadenylate-binding protein At2g36660 OS=Arabidopsis thaliana GN=At2g36660 PE=2 SV=1MATVHAALHAADASSSGSSSPVTASLYVGDLHPSVTEGILYDAFAEFKSLTSVRLCKDASSGRSLCYGYANFLSRQDANLAIEKKNNSLLNGKMIRVMWSVRAPDARRNGVGNVFVKNLPESVTNAVLQDMFKKFGNIVSCKVATLEDGKSRGYGFVQFEQEDAAHAAIQTLNSTIVADKEIYVGKFMKKTDRVKPEEKYTNLYMKNLDADVSEDLLREKFAEFGKIVSLAIAKDENRLCRGYAFVNFDNPEDARRAAETVNGTKFGSKCLYVGRAQKKAEREQLLREQFKEKHEEQKMIAKVSNIYVKNVNVAVTEEELRKHFSQCGTITSTKLMCDEKGKSKGFGFVCFSTPEEAIDAVKTFHGQMFHGKPLYVAIAQKKEDRKMQLQVQFGNRVEARKSSSSASVNPGTYAPLYYTNTHPGMVYQSYPLMWKSANMIGSSYPNSEAVTYPPMVANAPSKNRQNRIGKLDRNAVSYVPNVYQSTQMLPLSRDFSKQQHSRTYGRGKEMKKSIQQRQSETVGMEMQLLGELLHPLVEKLEPQLANKITGMLLEMDKSELLLLLKSPEDLAVRVDEAFEVLKSSKTNLTAPNTHRSDYLASGIAGVSIK>sp|Q54BM2|PAP1A_DICDI Polyadenylate-binding protein 1-A OS=Dictyostelium discoideum GN=pabpc1A PE=1 SV=1MATNFTPISSSLYVGDLLPEVSEQHLFEIFNQVGLVSNIRVCRDTNTRRSLSYAYVNYYNGADAERALDTLNNTPIRGKACRIMWSQRDPSLRKSGVGNVFIKNLDKGIDHKALYDTFSAFGNILSCKVVTDDGNSSKGFGFVHYETQESADKAIAKVNGMMINGQKVFVGPFKSSKERGQPTEVKFTNVFFKNLSEDVGPDQLKELLQQYGEITNITIMADDKGKSKGFGFANFESAEAAKNVVENENGKIFHGKPIYAGRAQKKIEREAELKHTFETKYQGVNLYIKNIDDSIDNDKLREVFSQFGTITSAIVMKDDKATTSKGFGFVCYTAPDEATRAVTEMNGRMIGTKPLYVALAQRKDIRRAQLEMQHQQKFKTGIRQQMPPTYGSGPVFFTPAPVNPQVVYQQMMPRPRNWNGQPVGVPQGQYANMNYARGQPRQNGPRQNGGQPRQNGPRPDVSGAQPIPVQQQTTLDVAQTQQAASSAESALNLQSIINLPSRDQQNVALGEHLYPLIHNSQPDLAGKITGMLLDSLPVEELFTLTQRQDLLADKIREALEVLGSN>sp|P04147|PABP_YEAST Polyadenylate-binding protein, cytoplasmic and nuclear OS=Saccharomyces cerevisiae (strain ATCC 204508 / S288c) GN=PAB1 PE=1 SV=4MADITDKTAEQLENLNIQDDQKQAATGSESQSVENSSASLYVGDLEPSVSEAHLYDIFSPIGSVSSIRVCRDAITKTSLGYAYVNFNDHEAGRKAIEQLNYTPIKGRLCRIMWSQRDPSLRKKGSGNIFIKNLHPDIDNKALYDTFSVFGDILSSKIATDENGKSKGFGFVHFEEEGAAKEAIDALNGMLLNGQEIYVAPHLSRKERDSQLEETKAHYTNLYVKNINSETTDEQFQELFAKFGPIVSASLEKDADGKLKGFGFVNYEKHEDAVKAVEALNDSELNGEKLYVGRAQKKNERMHVLKKQYEAYRLEKMAKYQGVNLFVKNLDDSVDDEKLEEEFAPYGTITSAKVMRTENGKSKGFGFVCFSTPEEATKAITEKNQQIVAGKPLYVAIAQRKDVRRSQLAQQIQARNQMRYQQATAAAAAAAAGMPGQFMPPMFYGVMPPRGVPFNGPNPQQMNPMGGMPKNGMPPQFRNGPVYGVPPQGGFPRNANDNNQFYQQKQRQALGEQLYKKVSAKTSNEEAAGKITGMILDLPPQEVFPLLESDELFEQHYKEASAAYESFKKEQEQQTEQA>sp|Q6FKG4|PABP_CANGA Polyadenylate-binding protein, cytoplasmic and nuclear OS=Candida glabrata (strain ATCC 2001 / CBS 138 / JCM 3761 / NBRC 0622 / NRRL Y-65) GN=PAB1 PE=3 SV=1MADITEKTAEQLENLSLQDKQEGTNEENQSETVSASLYVGDLDPSVSEAHLYDIFSPIGAVSSIRVCRDAITKTSLGYAYVNFNDHDAAKTAIEKLNFTPIKGKLCRIMWSQRDPSLRKKGAGNIFIKNLHPDIDNKALYDTFSVFGNILSSKVATDETGKSKGFGYVHFEEDESASEAIDALNGMLLNGQEIYVGPHLSKKERESKFEEMKANFTNVYIKNINTETTDKEFEELVAKFGKTDSVVLERTPEGENKGFGFVNFVNHEDAVKCVEELNNTEFKGQPLYVNRAQKKYERQQELKKQYEATRMEKMAKYQGINLFIKNLDDSIDDKKLEEEFAPYGTITSAKVMTTENGKSKGFGFVCFSTPEEATKAITEKNQQIVAGKPLYVAIAQRKDVRRSQLAQQIQARNQMRFQQASAAAAAAAAAGMPGQFMPPMFYGVMPPRGVPFNGPNPQMANMGAMPKNGMPPHQFRNGPVYGVPPQGGFARNGPAANQFYQQKQRQALGEELYKRIFSRTNDEEAAGKITGMILDLPPQEVVPLLENDELFEQHFKEASAAYESFKQEQQQPQGEEAQQA>sp|Q74ZS6|PABP_ASHGO Polyadenylate-binding protein, cytoplasmic and nuclear OS=Ashbya gossypii (strain ATCC 10895 / CBS 109.51 / FGSC 9923 / NRRL Y-1056) GN=PAB1 PE=3 SV=1MSDITDKTAEQLEQLKIEEQTAPTTTESETPKVETSGASLYVGELEPTVSEALLYDIFSPIGSVSSIRVCRDAITNTSLGYAYVNFHDHEAGPKAIEQLNYTLIKGKPCRIMWSQRDPSLRKKGSGNIYIKNLHPAIDNKSLHETFSTFGNILSCKVATDENGVSRGFGFVHFENESDARDAIEAVDGMLMNDQEVYVALHVSKKDRQSKLEEVKAKFTNVYVKNIDQETSQEEFEELFGKYGKITSAVLEKDSEGKLRGFGFVNFEDHAAAAKAVDELNELEFKGQKLYVGRAQKKYERLQELKKQYEAARLEKLAKYQGVNLFVKNLDDSIDDEKLKEEFAPFGTITSAKVMRDETGNSRGFGFVCFSTPEEATKAITEKNQQIVAGKPLYVAIAQRKEVRRNQLAQQIQARNQMRFQHANAAAAAAVAGLPGQFMPPPMYYGGIPPRVPFQGPNPQMAGMPKNGAMPPQQFGRPGPMYGGFAPQGQFPRNGQQQQFYQQKQRQALGEQLYQKVFAKTQDDEAAGKITGMILDLPPQQVIQLLENDELLEQHFQEAHAAYQKFKEDQEAQAAAAAAAAADARE>sp|Q6CSV3|PABP_KLULA Polyadenylate-binding protein, cytoplasmic and nuclear OS=Kluyveromyces lactis (strain ATCC 8585 / CBS 2359 / DSM 70799 / NBRC 1267 / NRRL Y-1140 / WM37) GN=PAB1 PE=3 SV=1MSDITEKTAEQLENLQINDDQQPAQSASAPSTSASESEASSVSKVENNNASLYVGELDPNITEALLYDVFSPLGPISSIRVCRDAVTKASLGYAYVNYTDYEAGKKAIQELNYAEINGRPCRIMWSERDPAIRKKGSGNIFIKNLHPAIDNKALHETFSTFGEVLSCKVALDENGNSRGFGFVHFKEESDAKDAIEAVNGMLMNGLEVYVAMHVPKKDRISKLEEAKANFTNIYVKNIDVETTDEEFEQLFSQYGEIVSAALEKDAEGKPKGFGFVNFVDHNAAAKAVEELNGKEFKSQALYVGRAQKKYERAEELKKQYEQYRLEKLAKFQGVNLFIKNLDDSIDDEKLKEEFAPYGTITSARVMRDQEGNSKGFGFVCFSSPEEATKAMTEKNQQIVAGKPLYVAIAQRKDVRRSQLAQQIQARNQIRFQQQQQQQAAAAAAGMPGQYMPQMFYGVMAPRGFPGPNPGMNGPMGAGIPKNGMVPPPQQFAGRPNGPMYQGMPPQNQFPRHQQQHYIQQQKQRQALGEQLYKKVSAKIDDENAAGKITGMILDLPPQQVIQLLDNDEQFEQQFQEALAAYENFKKEQEAQA>sp|Q5AI15|PABP_CANAL Polyadenylate-binding protein, cytoplasmic and nuclear OS=Candida albicans (strain SC5314 / ATCC MYA-2876) GN=PAB1 PE=3 SV=1MSAAETNQLQESMEKLNIGSTTEEQSAAAATTTADQSAEEQGESSGVAENSASLYVGELNPSVNEATLFEIFSPIGQVSSIRVCRDAVSKKSLGYAYVNYHKYEDGEKAIEELNYNPIEGRPCRIMWSQRDPSARRSGDGNIFIKNLHPAIDNKALHDTFSAFGKILSCKVATDEFGQSKCFGFVHYETAEAAEAAIENVNGMLLNDREVFVGKHISKKDRESKFEEMKANFTNIYVKNIDLNYSEESFEKLFSPFGKITSIYLEKDQDGKSKGFGFVNFEDHESAVKAVEELNDKEINGQKIYVGRAQKKRERLEELKKQYEAVRLEKLAKYQGVNLFVKNLDDTIDSEKLEEEFKPFGTITSAKVMVDEAGKSKGFGFVCFTTPEEATKAITEMNTRMINGKPLYVALAQRKDVRRSQLEQQIQARNQMRMQNAAAGGLPGQFIPPMFYGQQGFFPPNGRGNAPYPGPNPQMMMRGRGQPFPEQWPRPGPNGQPVPVYGIPPQFQQDFNGQNMRPQQQQQQQPRGGYYPNRNQTSKRDLAAIISSVPQDQQKRILGEELYPKIVATGKAQEPEAAGKITGMMLGLENQEILDLLDDDELFNNHFEDALTAFEEYKKSEAAGNAEEQA>sp|Q6BI95|PABP_DEBHA Polyadenylate-binding protein, cytoplasmic and nuclear OS=Debaryomyces hansenii (strain ATCC 36239 / CBS 767 / JCM 1990 / NBRC 0083 / IGC 2968) GN=PAB1 PE=3 SV=2MSAADANQVQESLEKLNLDSAPVASTEETEQTASGETEEAADSAQVSDTSASLYVGELNPSVNEALLFEIFSPIGQVASIRVCRDAVTKKSLGYAYVNFHKFEDGEKAIEDLNYSLIEGRPCRIMWSQRDPSLRRNGDGNIFIKNLHPAIDNKALHDTFTAFGKILSCKVATDDMGISKCFGFVHYETAEAAEAAIENVNGMLLNDREVYVGKHISKKDRESKFEEMKANFTNVYAKNIDLDFSEEEFKKLFEAYGKITSIYLEKDHEGKSKGFGFVNFENHESAVKAVDELNDKEINGQKIYVGRAQKKRERLEELKKQYENTRLEKLSKYQGVNLFIKNLDDTIDSEKLEEEFKPFGSITSARVMVDETGKSKGFGFVCFSSPEEATKAITEMNQRMIYGKPLYVALAQRKDVRRSQLEQQIQARNQMRMQNAAATGGIPGQFIPPMFYGQQPGFFPPNGRGNGPFPGPNPQMMMPRGQIPPPQGQWPRPGPNGQPVPVYGMPPVYGGDFNNGANGGRQQRGYYPNRNQNQKGRQQKDLAAIIANAPADQQKRILGEELYPKIVSTGKAQEPEAAGKITGMMLDLDNEEILALLEDDELFNTHFEDALTAFEEYKKSSEAATAEN>sp|A5DW14|PABP_LODEL Polyadenylate-binding protein, cytoplasmic and nuclear OS=Lodderomyces elongisporus (strain ATCC 11503 / CBS 2605 / JCM 1781 / NBRC 1676 / NRRL YB-4239) GN=PAB1 PE=3 SV=1MSAAETNQVQESLEKLNLDSSSSPAAGGATTATTTNNAESSDATSSSVPADSAEEQGESSGIAENSASLYVGELNPSVNEATLFEIFSPIGQVASIRVCRDAVSKKSLGYAYVNYHKLEDGEKAIEELNYTPVEGRPCRIMWSQRDPSARRSGDGNIFIKNLHPAIDNKALHDTFSAFGKILSVKVATDDLGQSKCFGFVHYETEEAAQAAIESVNGMLLNDREVYVGKHVSKKDRESKLEEMKANYTNIYVKNIDLAYTEKEFEELFAPFGKITSIYLEKDAEGKSKGFGFVNFEEHEAAAKAVEELNDKEINGQKIYVGRAQKKRERTEELKKQYEAVRLEKLSKYQGVNLFVKNLDEQIDSEKLEEEFKPFGTITSSKVMVDDAGKSKGFGFVCFSTPEEATKAITEMNQRMVNGKPLYVALAQRKDVRRSQLEQQIQARNQMRMQNAAAAGGLPGQFMPPMFYGQQGFFPPNGRGNAPFPGPNPQMMMRGRGQPFPEQWPRPGPNGQPVPVYGMPPQFQDFNGQNMRPQQQQQQQQQQQQQQQQQRGYYPNRPAGGNVPAKDLAALIANAPLEAQKRILGEELYQRIVATGKAQEPEAAGKITGMMLGLENQEILDLLDDEELFNNHFEEALNAFEEYKNSEGANAATGAPAPSEEA>sp|A3LXL0|PABP_PICST Polyadenylate-binding protein, cytoplasmic and nuclear OS=Scheffersomyces stipitis (strain ATCC 58785 / CBS 6054 / NBRC 10063 / NRRL Y-11545) GN=PAB1 PE=3 SV=1MSAADANQLQESLEKLNLDSAPAAAEEEAVAAESAPAGEEGADSANVAESTASLYVGELNTSVNEATLFEIFSPIGQVSSIRVCRDAVSKKSLGYAYVNYHKMEDGEKAIEELNYSPIEGRPCRIMWSQRDPSARRSGDGNIFIKNLHPAIDNKALHDTFSTFGKILSCKVATDDMGQSKCFGFVHYETAEAAEAAIENVNGMLLNDREVFVGKHISKKDRESKFEEIKANFTNIYVKNIDLEYSEEDLKKLFTPYGAITSIYLEKDAEGKSKGFGFVNYEGHEAAVKAVEELNDKEINGQKIYVGRAQKKRERMEELKKQYENTRLEKLSKYQGVNLFIKNLDDTIDSEKLEEEFKPFGTITSARVMVDETGKSKGFGFVCFSSPEEATKAITEMNQRMFFGKPLYVALAQRKDVRRSQLEQQIQARNQMRMQNAAATGGIPGQFIPPMFYGQQGFFPPNGRGNAPFPGPNPQMIMRRGQPFGGPEQWPRPGPNGQPVPVYGIPPQAYSDFNGQNIRQQRGYYPNRNQNKGRQQRDLAAIIASAPPDQQKRILGEELYPKIVATGKAQEPEAAGKITGMMLDLDNQEILALLEDDELFTNHFEDALTAFEEYKNSEAAAPVAPAAPAEPQA>sp|A5DM21|PABP_PICGU Polyadenylate-binding protein, cytoplasmic and nuclear OS=Meyerozyma guilliermondii (strain ATCC 6260 / CBS 566 / DSM 6381 / JCM 1539 / NBRC 10279 / NRRL Y-324) GN=PAB1 PE=3 SV=2MSDLQESLEKLSINEKAPQAPADDATPSNTTTLEKESSESAAAAAGEGAGEEGEEASASLYVGELNPSVNEALLFEIFSPIGQVSSIRVCRDAVTKKSLGYAYVNFHKHADGSRAIEELNYSLVDGRPCRIMWSQRDPSLRRNGDGNIFIKNLHPAIDNKALHDTFSAFGRILSCKVATDELGQSKCFGFVHYETAEAAEAAIENVNGMLLNDREVFVGKHVSKRDRESKFEEMKANFTNVYVKNLAPEVDNAKFEEIFKPFGPVTSVHLETDQEGKSRGFGFVNFENHESALNAVKEMNDKEIDGQKLYVGRAQKKRERLDELKRLYESTRLEKLSKYQGVNLFVKNLDDSIDSEKLEEEFKPFGTITSARVMVDDAGKSKGFGFVCFSSPEEATKAITEMNQRMIQGKPLYVALAQRKDVRRSQLEQQIQARNQMRMQNAAAAAGMPGQFMSPMFYGQQPGFFPPNGRGGAQGPFPPNPQMMMPRGGQMPPPQGQWPRAGPNGQPVPVYGMPPVYGGEFNGPNGQRQQRGAYPPNRNQKGGRPQRDLAAIISTVPVDQQKRILGEELYPKIVATGKAQEPEAAGKITGMMLDLENEEILALLEDDELFENHFEDALTAFEEYKKGEQAE>sp|Q6CDH3|PABP_YARLI Polyadenylate-binding protein, cytoplasmic and nuclear OS=Yarrowia lipolytica (strain CLIB 122 / E 150) GN=PAB1 PE=3 SV=1MTLENKAEASPATKEETTTEAAPAEGEAKTESSEEKGSKEDQGDNASLYVGELDPSVTEAMLFEIFNPIGPVTSVRVCRDAITRRSLGYAYVNFHNQADGIRALEELNYSPIKERPCRIMWSQRDPALRKTGAGNIYIKNLDPAIDNKALHDTFSAFGQILSCKIATDEFGNSRGFGFVHYESAESAESAIQHVNGMLLNDKKVFVGPHVPKSDRMQSFEEQKNSFTNVFIKNLGTEITEAEFEELVNKFGETSSVHLSTNDEGKPTGFGFVDYKEHDVAVKAIDGLSETEFKGNKLFAGRAKKKYERADELRKQYEASRLEKLNKYQGVNLYIKNLDDTIDDDKLRAEFAPHGTITSAKVMVDEAGKSKGFGFVCYSSPEEATKAVTEMNHRLVAGKPLYVVLAQRKDVRRSQLQQQIQAKNQMRLQQQAAAGGLPGQYMGNPGVFYPGQPGFMPPGRGGMPFGANPQMMMRPPMPPQNQFPPRGVPGGPNMYGAPPQGYQQGGFPPQGPMRGGQPPRSGQPGPQGQFRGAPRRKDGESRVADSISNALENAPEEQHKQLVGEALYPKVLAEKAIDGNAEFAGKITGMLLEMPIKEILEVIDDEEGLQAQINDAITAYNEYLNSQKEE>sp|P31209|PABP_SCHPO Polyadenylate-binding protein, cytoplasmic and nuclear OS=Schizosaccharomyces pombe (strain 972 / ATCC 24843) GN=pab1 PE=1 SV=2MPSTDLKKQADAAVESDVNTNNEAVESSTKEESSNTPSTETQPEKKAEEPEAAAEPSESTSTPTNASSVATPSGTAPTSASLYVGELDPSVTEAMLFELFNSIGPVASIRVCRDAVTRRSLGYAYVNFHNMEDGEKALDELNYTLIKGRPCRIMWSQRDPSLRKMGTGNVFIKNLDPAIDNKALHDTFSAFGKILSCKVAVDELGNAKGYGFVHFDSVESANAAIEHVNGMLLNDKKVYVGHHVSRRERQSKVEALKANFTNVYIKNLDTEITEQEFSDLFGQFGEITSLSLVKDQNDKPRGFGFVNYANHECAQKAVDELNDKEYKGKKLYVGRAQKKHEREEELRKRYEQMKLEKMNKYQGVNLFIKNLQDEVDDERLKAEFSAFGTITSAKIMTDEQGKSKGFGFVCYTTPEEANKAVTEMNQRMLAGKPLYVALAQRKEVRRSQLEAQIQARNQFRLQQQVAAAAGIPAVQYGATGPLIYGPGGYPIPAAVNGRGMPMVPGHNGPMPMYPGMPTQFPAGGPAPGYPGMNARGPVPAQGRPMMMPGSVPSAGPAEAEAVPAVPGMPERFTAADLAAVPEESRKQVLGELLYPKVFVREEKLSGKITGMLLEMPNSELLELLEDDSALNERVNEAIGVLQEFVDQEPGFTE>sp|Q7S6N6|PABP_NEUCR Polyadenylate-binding protein, cytoplasmic and nuclear OS=Neurospora crassa (strain ATCC 24698 / 74-OR23-1A / CBS 708.71 / DSM 1257 / FGSC 987) GN=pab-1 PE=3 SV=1MAATSTAAVDQLAADLGNTSLDNKAAAPAPIDTSAVPEAQAEGAEAAPTPTAAPHPQASASLYVGELDPSVTEAMLFELFSQIGSVASIRVCRDAVTRRSLGYAYVNYNTTADGEKALEELNYTLIKGRPCRIMWSQRDPALRKTGAGNIFIKNLDAAIDNKALHDTFAAFGNILSCKVAQDEHGNSKGYGFVHYETDEAASQAIKHVNGMLLNEKKVYVGHHIPKKDRQSKFEEMKANFTNVYVKNINNEVTDEEFRELFAKFGEVTSSSLARDQEGKSRGFGFVNFTTHEAAAQAVDELNGKDFRGQDLYVGRAQKKHEREEELRKSYEAARLEKANKYQGVNLYIKNLGDDVDDDKLRAMFSEYGPITSAKVMRDSLIEGSEEKDEKDKENKKEGETKEEEQNEGSEKKTEKKGDRKLGKSKGFGFVCFSNPDDATKAVTEMNQRMVDGKPLYVALAQRKDVRKSQLEASIQARNQLRMQQAAAQAGMPQQYMQAPVYYAGQQPGFMPAPGGRGVPFPQGGIVPGVQGGRPGQYPYQQGGRGGVPPQQMPPMGYPINQFGPGAFPPNTPQYMAAMGQVGALGGGRGGPAGRGPQGIPAGIPQGLQGGPAVPGYPPAGRPQNGGGRGTPRGNANFMAAGRGASPIPGAPADLSAGSFLQAQLATTQDPQAQKQIIGENLFPKIQAIQPALAGKITGMLLEMDNAELINLFEDDNALNVKVQEALAVYDEYLKTQGQQPTQQPAEANGEQPKAEEQKPEEQKA>sp|Q5B630|PABP_EMENI Polyadenylate-binding protein, cytoplasmic and nuclear OS=Emericella nidulans (strain FGSC A4 / ATCC 38163 / CBS 112.46 / NRRL 194 / M139) GN=pab1 PE=2 SV=2MSAETSTTPAPAENTNGTPDNAPAPEVTAVEAPATTSQPHSASLYVGELDPSVTEAMLYELFSSIGQVASIRVCRDAVTRRSLGYAYVNYNDTAHGERALDELNYTLIKGKPCRIMWSQRDPALRKTGQGNVFIKNLDSAIDNKALHDTFAAFGNILSCKVAQDEFGVSKGYGFVHYETAEAANNAIKHVNGMLLNDKKVFVGHHISKKDRQSKFEEMKANFTNIYIKNIDPEVEDEEFRKLFEKFGEITSATLSRDSEGKSRGFGFVNFSTHESAQAAVEEMNDKEVRSQKLYVGRAQKKHEREEELRKQYEAARMEKASKYQGVNLYVKNLTDDVDDDKLRELFGPYGTITSAKVMRDTAPVETATPESETKESANKENEKAAEGEKEPAAEEKEKEEKKEAEQKPEKKPLGKSKGFGFVCFSSPDEASKAVTEMNQRMVNGKPLYVALAQRKDVRRSQLEASIQARNNIRQQQAAAAAGMGQAYMAPAVFYGPGQQGFIPGAQRGGMFPPQPGMMMGMPGRPGQYPGPFPGQQGGRGVGPNQQIPPNFQGLPMGAMQGPGIPNGMGYPMVQGQFGGGRGRGQVPGMGGPMRGGYGGGRGGVPLGGQMRPGQGGRGQAVGQPGPETPVGVLTAQALSAAPPQQQKQMLGEALYPKIQATQPELAGKITGMLLEMDNTELLGLLEDDEALRAKVDEALSVYDEYMKNKSDEPAAEKPKEAAQEAPAEENKA>sp|Q0CR95|PABP_ASPTN Polyadenylate-binding protein, cytoplasmic and nuclear OS=Aspergillus terreus (strain NIH 2624 / FGSC A1156) GN=pab1 PE=3 SV=1MSAEASTTPAAETPVNGTPETSTTPAAPAAEATAAETAAPSTSQPHSASLYVGELDPSVTEAMLYELFSSIGQVASIRVCRDAVTRRSLGYAYVNYNNTADGERALEDLNYTLIKGKPCRIMWSQRDPALRKTGQGNVFIKNLDAAIDNKALHDTFAAFGNILSCKVAQDEFGNSKGYGFVHYETAEAANNAIKHVNGMLLNDKKVFVGHHISKKDRQSKFEEMKANFTNVYIKNLDQEISEEEFRQMFEKFGEITSATLSRDQEGKSRGFGFVNYSTHDSAQAAVDEMNDKEVKGQKLYVGRAQKKHEREEELRKQYEAARLEKASKYQGVNLYVKNLTDDIDDEKLREMFAPYGTITSAKVMRDTNIERTQTPDSDKEKKEESKEEKPEAAEKTEEAAKESGDDQDKENKKSDKKVLGKSKGFGFVCFSSPDEASKAVTEMNQRMINGKPLYVALAQRKDVRRSQLEASIQARNTIRQQQAAAAAGMPQPYMQPAVFYGPGQQGFIPGGQRGGLPFAPQPGMMMGVPGGRPGQYPGPFPGQQGGRGMGPNQQIPPNFAQGIPMGAMGPGGIPNGMGYPQMGQVQFGRGAGGRGQVPGMPMGQGMRGPGYGQGRGGVPVQGQMRPGQGGRGQNAQPAAGRGEEAPAAGLTAQSLAAAPAPQQKQMLGEALYPKIQAQQPELAGKITGMLLEMDNTELLSLLEDDEALRAKVDEALSVYDEYMKNKGTEGEAAGEAPKPKEAATEESTEENKS>sp|A4QUF0|PABP_MAGO7 Polyadenylate-binding protein, cytoplasmic and nuclear OS=Magnaporthe oryzae (strain 70-15 / ATCC MYA-4617 / FGSC 8958) GN=PAB1 PE=3 SV=1MAAPSNTAAVDQLTSDLANANMNGGEKTTVNTNVGASFTGEEIDTAGPTPSSAAPHPQASASLYVGELDPSVTEAMLFELFSQIGSVASIRVCRDAVTRRSLGYAYVNYNTTADGEKALEELNYTLIKGRPCRIMWSQRDPALRKTGQGNVFIKNLDVAIDNKALHDTFAAFGNILSCKVAQDENGNSKGYGFVHYETDEAASQAIKHVNGMLLNEKKVYVGHHIPKKDRQSKFDEMKANFTNIYVKNINPEVTDDEFRTLFEKYGDVTSSSLARDQETGKSRGFGFVNFTSHEDASKAVQELNEKEFHGQNLYVGRAQKKHEREEELRKSYEAARQEKASKYQGVNLYIKNLDDEVDDEKLRQLFSEFGPITSAKVMRDSITEPGEEGESKEGEESEKNKENKPEEKEGDDSKPEEKEGEDSKSKSKLGKSKGFGFVCFANPDDATKAVAEMNQRMVNNKPLYVALAQRKDVRKNQLEQSIQARNQLRMQQAAAAAGMPQQYMQAPVFYGPGSQPGFMPPAGGRGMPYPQGGMPMQPGRPGQFPAGFAAQQGGRGAMPQGIPPMYGLPGQFPPQGPFPQPNNPQFLAAMQQIQQSALAGGRGGPAGRGPMQGGVPVPGMPGGAGLPGFPPNARQQGPGAGRGAAAGRAPAGAPAGARGAGAPEGLQGQLAAVADNPGQQKQILGEAIFPKIQAIHPELAGKITGMLLEMDNTELVALVENDGALRSKVDEALAVYDDYVRQQGDGEGAQAPSKEEKTEEKA>sp|A1CRM1|PABP_ASPCL Polyadenylate-binding protein, cytoplasmic and nuclear OS=Aspergillus clavatus (strain ATCC 1007 / CBS 513.65 / DSM 816 / NCTC 3887 / NRRL 1) GN=pab1 PE=3 SV=1MSAEVSTTPAADNVNGTPEATNAAATSAPEVTAVESSSPTSPNNNNQPHSASLYVGELDPSVTEAMLYELFSSIGQVASIRVCRDAVTRRSLGYAYVNYNNTADGERALEDLNYTLIKGKPCRIMWSQRDPALRKTGQGNVFIKNLDSAIDNKALHDTFAAFGNILSCKVAQDEFGNSKGYGFVHYETAEAANNAIKHVNGMLLNDKKVFVGHHISKKDRQSKFDEMKANFTNIYIKNIDPDVTEEEFRKIFEQFGEITSATLSRDPEGKSRGFGFVNYSTHESAQAAVDEMHDKEVKTQKLYVGRAQKKHEREEELRKQYEAARLEKASKYQGVNLYVKNLTDDVDDEKLRELFGPYGTITSAKVMRDSTPAERTETPDSEKEKEVNKENEKKEDEEKAAEEKPKESDEEKKDETKKSDKKLLGKSKGFGFVCFSSPDEASKAVTEMNQRMVNGKPLYVALAQRKDVRRSQLEASIQARNTIRQQQAAAAAGMPQPYMQPAVFYGPGQQGFIPAGQRGGMPFAPQPGMVMGIPGGRPGQYPGPFPGQQGGRGMGPNQQMPPNFQGIPMGAMQGPGGIPNGMGYPQAMGQVQFGRGGGRGQVPGMPMGQGMRGPGYQGRGGPQGGPRPQGGRGQNAAAQPAAGREEAPAGALTAQALNAAAPPQQKQMLGEALYPKIQAQQPELAGKITGMLLEMDNTELLGLLEDDDALRAKVDEALSVYDEYMKNKGEGEAPADADKPKEAAKETATEENKS>sp|Q4P8R9|PABP_USTMA Polyadenylate-binding protein, cytoplasmic and nuclear OS=Ustilago maydis (strain 521 / FGSC 9021) GN=PAB1 PE=3 SV=1MSSTESPVPAAAAPAEAVPASTPAPAAEQPAVGNGEQRNNADAANNTSLYVGELDPSVTEAMLFEIFSMIGTVASIRVCRDAVTRRSLGYAYVNFLNAADGERAMEQLNYSLIRNRPCRIMWSQRDPALRRTGQGNIFIKNLDAGIDNKALHDTFAAFGNILSCKVATNETGSLGYGFVHYETAEAAEAAIKHVNGMLLNDKKVYVGHHIPRKERQAKIEETRANFTNVYAKNVDPEVTDDEFEKLFTKFGKITSCVLQRDEDGKSKGFGFVNFEDHNEAQKAVDELHDSDFKGQKLFVARAQKKSEREEELRRSYEAAKNEKLAKFQGVNLYLKNIPESYDDERLREEFAPFGAITSCKIMRAPSGVSRGFGFVCYSAPEEANKAVSEMNGKMLDNRPLYVALAQRKDVRRQQLEAQIMQRNQLRLQQQAAAQGMGYPGPGMYYPQPGAFPGQPGGMVPRPRYAPAGMMPQGMPMAPYGQPGQFPAGMMPQGYRPARPPRGAPNAAGGPAPPAGARPPTGVNGAPRPAGQPVPGQPMPRGPAARPAGRPEADQPGALTAAALAKASPEEQKQMLGEAIYPKVAASQPELAGKLTGMILELPVTELLHLLEESEALDAKVNEALEVLKEYQQNDSAGAEAEANAEAPKTEA>sp|A1D4K4|PABP_NEOFI Polyadenylate-binding protein, cytoplasmic and nuclear OS=Neosartorya fischeri (strain ATCC 1020 / DSM 3700 / FGSC A1164 / NRRL 181) GN=pab1 PE=3 SV=1MSAEVSTTPAADNTVNGTPEATNPAATSAPEVTAVESASPSATPSANQPHSASLYVGELDPSVTEAMLYELFSSIGQVASIRVCRDAVTRRSLGYAYVNYNNTADGERALEDLNYTLIKGKPCRIMWSQRDPALRKTGQGNVFIKNLDAAIDNKALHDTFAAFGNILSCKVAQDEFGNSKGYGFVHYETAEAANNAIKHVNGMLLNDKKVFVGHHISKKDRQSKFEEMKANFTNVYIKNIDQEVTDEEFRKMFEKFGEITSATLSRDQEGKSRGFGFVNFSTHDSAQAAVDEMNDKEIKGQKLYVGRAQKKHEREEELRKQYEAARLEKASKYQGVNLYVKNLTDDVDDEKLRELFSPFGTITSAKVMRDTVTAGETSESEKEKESNKENEKEGEEKTEEKPKESEEEAKKTEKKILGKSKGFGFVCFSSPDEASKAVTEMNQRMVNGKPLYVALAQRKDVRRSQLEASIQARNTIRQQQAAAAAGMPQPYMQPAVFYGPGQQGFIPAGQRGGMPFAPQPGMVMGIPGGRPGQYPGPFPGQQGGRGMGPNQQIPPNFQGIPMGAMQGPGGIPNGMGYPQMAQVQFGRGAGGRGQVPGMPMGQGMRGPGYGQGRGGAPVQGGPRPQGGRGQPAAAPPAAGREEVPATGGLTAQTLNAVPPPQQKQMLGEALYPKIQAQQPELAGKITGMLLEMDNTELLGLLEDEEALRAKVDEALSVYDEYMKNKGEGEAPAEPAKPKEDAAETATEENKS>sp|Q4WK03|PABP_ASPFU Polyadenylate-binding protein, cytoplasmic and nuclear OS=Neosartorya fumigata (strain ATCC MYA-4609 / Af293 / CBS 101355 / FGSC A1100) GN=pab1 PE=3 SV=1MSAEVSTTPAADNTVNGTPEATNAAATSAPEVTAVESASPSTTPSASQPHSASLYVGELDPSVTEAMLYELFSSIGQVASIRVCRDAVTRRSLGYAYVNYNNTADGERALEDLNYTLIKGKPCRIMWSQRDPALRKTGQGNVFIKNLDAAIDNKALHDTFAAFGNILSCKVAQDEFGNSKGYGFVHYETAEAANNAIKHVNGMLLNDKKVFVGHHISKKDRQSKFEEMKANFTNVYIKNIDQEVTDEEFRKMFEKFGEITSATLSRDQEGKSRGFGFVNFSTHDSAQAAVDEMNDKEIKGQKLYVGRAQKKHEREEELRKQYEAARLEKASKYQGVNLYVKNLTDDVDDEKLRELFSPFGTITSAKVMRDTVTTGETSESEKEKEKESNKENEKEGEEKTEEKPKESEEEPKKTEKKILGKSKGFGFVCFSSPDEASKAVTEMNQRMVNGKPLYVALAQRKDVRRSQLEASIQARNTIRQQQAAAAAGMPQPYMQPAVFYGPGQQGFIPAGQRGGMPFAPQPGMVMGIPGGRPGQYPGPFPGQQGGRGMGPNQQIPPNFQGIPMGAMQGPGGIPNGMGYPQMAQVQFGRGAGGRGQVPGMPMGQGIRGPGYGQGRGGAPVQGGPRPQGGRGQPAAAPPAAGREEVPATGGLTAQTLSAVPPPQQKQMLGEALYPKIQAQQPELAGKITGMLLEMDNNELLGLLEDEEALRAKVDEALSVYDEYMKNKGEGEAPAESAKPKEDAAETATEENKS>sp|Q0U1G2|PABP_PHANO Polyadenylate-binding protein, cytoplasmic and nuclear OS=Phaeosphaeria nodorum (strain SN15 / ATCC MYA-4574 / FGSC 10173) GN=PAB1 PE=3 SV=3MSEVANSTSPVQDGADANGAQINTNVPAASGDAPTPTTAAQQAHQNSASLYVGELDPSVTEAMLFELFSSIGQVASIRVCRDAVTRRSLGYAYVNYNSSEDGEKALEELNYTVIKGKPCRIMWSQRDPALRKTGQGNVFIKNLDHAIDNKALHDTFAAFGNILSCKVAQDELGNSKGYGFVHYETAEAANNAIKHVNGMLLNEKKVFVGHHIPKKERMSKFEEMKANFTNIYVKNIDLDVTDEDFRELFEKHGDITSASIARDDQGKSRGFGFVNYIRHEAAAVAVDHLNDIEFKGQKLYVGRAQKKHEREEELRKQYEAARLEKQSKYQGVNLYIKNLNDDVDDEKLRDMFTPFGTITSAKVILRDEEKKDEEEKEVKEEKKEDEKKEDEEAKEGSSSEQNGEDTKAGDKVTIKGEKKILGKSKGFGFVCFSNPDEATKAVTEMNQKMIEGKPLYVALAQRKDVRKNQLEATIQARNQLRMQQQQQQQFGGIPQMFIAPGQQPMMYPPGARGQMPFPAGMPGAQGGRGAGFPGGMPGQQGGRGGPNAQQMPPMYMPPGMAPGAFPPGPYMNQQYMQLAQAAQQAMGGRGGRGGPMPGMPGMPQAQIAGGPGIRGGQGGFPQGGRGAPGGRGQPPMPGFPQGGRPGGPGVDMSVLSAAAPGQQKQMLGEALYPKIHEMQPELAGKITGMLLEMDNSELINLTADESALRAKVDEAMSVYDEYVKNKEGDGEKEAPKEESKEEKA>sp|Q2GSX8|PABP_CHAGB Polyadenylate-binding protein, cytoplasmic and nuclear OS=Chaetomium globosum (strain ATCC 6205 / CBS 148.51 / DSM 1962 / NBRC 6347 / NRRL 1970) GN=PAB1 PE=3 SV=1MAAPVAPGAVDQLAADLGNTSLGGGDNRAAPAINTNVAPGEYQTADPDTAGPTPSSAAPHPQSSASLYVGELDPSVTEAMLFELFSQIGSVASIRVCRDTITRRSLGYAYVNYNSTSDGEKALEELNYTLIKGRPCRIMWSQRDPALRKTGQGNVFIKNLDVAIDNKALHDTFAAFGNILSCKVAQDENGNSKGYGFVHYETDEAAAQAIKHVNNMLLNEKKVYVGYHIPKKDRQSKFEEMKANFTNIYVKNISLEATDEEFRDLFAKYGDVTSSSLARDSEGKSRGFGFVNFTTHECAAKAVEELNGKEFRGQDLYVGRAQKKHEREEELRKSYEAARLEKANKYQGVNLYIKNLADDIDDDKLRQMFSEYGPITSAKVMRDAVTEGSAEEETEGKDKENKKEGEQAAEAEGEAEGAEKKTEKKGDRRLGKSKGFGFVCFSNPDDATKAVAEMNQRMIEGKPLYVALAQRKDVRKNQLEASIQARNQLRMQQAAAQAGLPQQYMQTPVYYAPGQQPNFMPPGGRGMPFPQGGLGMPAVQGGRPGQFPPYAQQGGRGGMPPQQLPIYPLGQFPPGAYPQPNNPQFLAAIQQVQQQAAALGNGRGGPGGPGGRGMQGMPVPQGMPGGPGMAGFPPNGRPQNGNMGGRGGPGRGGNFAAGRGAPPAGPLAAGGELNASSLLQSQLTATNNPQQQKQILGENLFPKIQALQPDLAGKITGMLLEMDNAELVNLLEDEAALVAKVNEAMAVYDEYVKSQQGPGQGPAPTQGEAEAEKPKEEKAEEKA>sp|A2Q848|PABP_ASPNC Polyadenylate-binding protein, cytoplasmic and nuclear OS=Aspergillus niger (strain CBS 513.88 / FGSC A1513) GN=pab1 PE=3 SV=1MSADVSTTPAAENVNGAAEASPAPAAAAPSATTPEVTAVENSTPAPAANQPHSASLYVGELDPSVTEAMLYELFSSIGQVASIRVCRDAVTRRSLGYAYVNYNNTADGERALEDLNYTLIKGKPCRIMWSQRDPALRKTGQGNVFIKNLDSAIDNKALHDTFAAFGNILSCKVAQDEFGNSKGYGFVHYETAEAANNAIKHVNGMLLNDKKVFVGHHISKKDRQSKFEEMKANFTNVYIKNLDSEIDDDEFRKMFEKFGEITSATLSRDQEGKSRGFGFVNFSTHESAQAAVEEMNDKEIRSQKLYVGRAQKKHEREEELRKQYEAARLEKASKYQGVNLYVKNLTDDIDDEKLRELFGPYGTITSAKVMRDTNVERDQSPDSAGKEKEADKENDKEATPEAEKAEKAEEKPSESSEEKDKEAKKSDKKPFGKSKGFGFVCFSSPDEASKAVTEMNQRMVNGKPLYVALAQRKDVRRSQLEASIQARNTIRQQQAAAAAGMPQPYMQPAVFYGPGQQGFIPGGQRGMAFPPQPGMVMGIPGGRPGQYPGPFPGQQGGRGMGPNQQIPPNFAQGIPMGMQGPGGIPNGMGYPQMAQVQFGRGAGGRGQVPGMPMGQGMRGGPGYGQGRGAPVQQGQMRPGQGGRGQNAAAPAGPQEGAAGGVNAQTLGAAPPAQQKQMLGEALYPKIQAQQPELAGKITGMLLEMDNTELLSLTRKPCAPRSMRPLAFTMST>sp|Q1DXH0|PABP_COCIM Polyadenylate-binding protein, cytoplasmic and nuclear OS=Coccidioides immitis (strain RS) GN=PAB1 PE=3 SV=1MSAETATNPPVDTTPGAAPESATNGSNANVAADTTAGEASQTTSSTTPTAQPHSASLYVGELDPSVTEAMLFELFSSIGQVASIRVCRDAVTRRSLGYAYVNYNNTADGERALEDLNYTLIKGRPCRIMWSQRDPALRKTGQGNVFIKNLDTAIDNKALHDTFAAFGNILSCKVAQDEFGNSKGYGFVHYETAEAAQNAIKHVNGMLLNDKKVFVGHHIAKKDRQSKFEEMKANFTNVYVKNIDQDTTEEEFRDLFEKFGEITSATLARDAESGKSRGFGFVNFTSHDNAAAAVEALNDKDFKGQKLYVGRAQKKHEREEELRKQYEAARIEKASKYQGVNLYIKNLSDDIDDEKLRELFSSYGTITSAKVMRDFAPESTSDSEKEAKKDSKEPETKEEEPKDEAGDNAENKDNKENKAESKKSEKKPLGKSKGFGFVCFSSPDEASKAVTEMNQRMVHGKPLYVALAQRKDVRRSQLEASIQARNTIRQQQAAAAAGMPQPFMQPAVFYGPGQQNFIPNQRGGMPFQQPGMVIPGMPGGRHGQFGGFPGQQGGRGMNPNQQIPPNAYGIGAQGLPMGMQGAGIPNGLNYPQMGQVQAPFGRGRGQAPSGQGMPPNVQGMGPGGQYGRGMPVQQGMGRPGQAGRGQGAPAQAVGQRDENASPNGLTLQVLNAAPPAQQKQMLGEAIYPKIQAQQPELAGKITGMLLEMDNAELLALVDDDAALKAKVDEALTVYDEYVKNKGGDSGEPAADANKSKDASQETAEETKS>sp|P0CP46|PABP_CRYNJ Polyadenylate-binding protein, cytoplasmic and nuclear OS=Cryptococcus neoformans var. neoformans serotype D (strain JEC21 / ATCC MYA-565) GN=PAB1 PE=3 SV=1MSAETATSPAPAAETPVAPAPATQTTPAEGAPTPAAAAPGGNTVSASLYVGELDPSVTEAMLFEIFNMIGPVASIRVCRDAVTRRSLGYAYVNYLNAADGERALEHLNYSLIKGQSCRIMWSQRDPALRKTGQGNIFIKNLDQSIDNKALHDTFAAFGDILSCKVGTDENGKSRGFAFVHYSTGEAADAAIKAVNGMLLNDKKVYVGHHVGKKERLSKVEELRAQFTNVYIKNVDLEVTDAEFEDLVKPFGPTISVALSRDEKGVSKGFGFVNYENHESARKAVDELNEKEVNGKKLYAGRAQTKSEREAELKKSHEEKRLENEAKSAGVNLYVKNLDDEWDDDRLRAEFEAFGTITSSKVMRDDSGVSRGFGFVCYSSPDEATKAVSEMNGKMIGTKPLYVALAQRKDVRRQALESQIAQRAQQRMQYGAGFPGMQGYMGQPMYGYPPMPGYGQPMPGMPPVRGPMMGYPGAPQNMMQSRPRFNPNGQPLPGGVPAYGMPPQVPYPGAPGYPVRPGGARIPAAPNANGPRNGGPSPVGAPQGLPAGSIPRGGQMPARPHEQAAPAPQAGRLDAQSLARAAPAEQKQMLGEALYPLIHETQPELAGKITGMLLEMDNAELLHLVESQPALQEKVDEALRVLAEWGKDEKPAADEGAEEPKKEEEETKEEEKKE>sp|Q9EPH8|PABP1_RAT Polyadenylate-binding protein 1 OS=Rattus norvegicus GN=Pabpc1 PE=2 SV=1MNPSAPSYPMASLYVGDLHPDVTEAMLYEKFSPAGPILSIRVCRDMITRRSLGYAYVNFQQPADAERALDTMNFDVIKGKPVRIMWSQRDPSLRKSGVGNIFIKNLDKSIDNKALYDTFSAFGNILSCKVVCDENGSKGYGFVHFETQEAAERAIEKMNGMLLNDRKVFVGRFKSRKEREAELGARAKEFTNVYIKNFGEDMDDERLKELFGKFGPALSVKVMTDESGKSKGFGFVSFERHEDAQKAVDEMNGKELNGKQIYVGRAQKKVERQTELKRKFEQMKQDRITRYQGVNLYVKNLDDGIDDERLRKEFSPFGTITSAKVMMEGGRSKGFGFVCFSSPEEATKAVTEMNGRIVATKPLYVALAQRKEERQAHLTNQYMQRMASVRAVPNPVINPYQPAPPSGYFMAAIPQTQNRAAYYPPSQIAQLRPSPRWTAQGARPHPFQNMPGAIRPAAPRPPFSTMRPASSQVPRVMSTQRVANTSTQTMGPRPAAAATAATPAVRTVPQYKYAAGVRNPQQHLNAQPQVTMQQPAVHVQGQEPLTASMLASAPPQEQKQMLGERLFPLIQAMHPSLAGKITGMLLEIDNSELLHMLESPESLRSKVDEAVAVLQAHQAKEAAQKAVNSATGVPTV>sp|P29341|PABP1_MOUSE Polyadenylate-binding protein 1 OS=Mus musculus GN=Pabpc1 PE=1 SV=2MNPSAPSYPMASLYVGDLHPDVTEAMLYEKFSPAGPILSIRVCRDMITRRSLGYAYVNFQQPADAERALDTMNFDVIKGKPVRIMWSQRDPSLRKSGVGNIFIKNLDKSIDNKALYDTFSAFGNILSCKVVCDENGSKGYGFVHFETQEAAERAIEKMNGMLLNDRKVFVGRFKSRKEREAELGARAKEFTNVYIKNFGEDMDDERLKELFGKFGPALSVKVMTDESGKSKGFGFVSFERHEDAQKAVDEMNGKELNGKQIYVGRAQKKVERQTELKRKFEQMKQDRITRYQGVNLYVKNLDDGIDDERLRKEFSPFGTITSAKVMMEGGRSKGFGFVCFSSPEEATKAVTEMNGRIVATKPLYVALAQRKEERQAHLTNQYMQRMASVRAVPNPVINPYQPAPPSGYFMAAIPQTQNRAAYYPPSQIAQLRPSPRWTAQGARPHPFQNMPGAIRPAAPRPPFSTMRPASSQVPRVMSTQRVANTSTQTMGPRPAAAAAAATPAVRTVPQYKYAAGVRNPQQHLNAQPQVTMQQPAVHVQGQEPLTASMLASAPPQEQKQMLGERLFPLIQAMHPSLAGKITGMLLEIDNSELLHMLESPESLRSKVDEAVAVLQAHQAKEAAQKAVNSATGVPTV>sp|P11940|PABP1_HUMAN Polyadenylate-binding protein 1 OS=Homo sapiens GN=PABPC1 PE=1 SV=2MNPSAPSYPMASLYVGDLHPDVTEAMLYEKFSPAGPILSIRVCRDMITRRSLGYAYVNFQQPADAERALDTMNFDVIKGKPVRIMWSQRDPSLRKSGVGNIFIKNLDKSIDNKALYDTFSAFGNILSCKVVCDENGSKGYGFVHFETQEAAERAIEKMNGMLLNDRKVFVGRFKSRKEREAELGARAKEFTNVYIKNFGEDMDDERLKDLFGKFGPALSVKVMTDESGKSKGFGFVSFERHEDAQKAVDEMNGKELNGKQIYVGRAQKKVERQTELKRKFEQMKQDRITRYQGVNLYVKNLDDGIDDERLRKEFSPFGTITSAKVMMEGGRSKGFGFVCFSSPEEATKAVTEMNGRIVATKPLYVALAQRKEERQAHLTNQYMQRMASVRAVPNPVINPYQPAPPSGYFMAAIPQTQNRAAYYPPSQIAQLRPSPRWTAQGARPHPFQNMPGAIRPAAPRPPFSTMRPASSQVPRVMSTQRVANTSTQTMGPRPAAAAAAATPAVRTVPQYKYAAGVRNPQQHLNAQPQVTMQQPAVHVQGQEPLTASMLASAPPQEQKQMLGERLFPLIQAMHPTLAGKITGMLLEIDNSELLHMLESPESLRSKVDEAVAVLQAHQAKEAAQKAVNSATGVPTV>sp|P61286|PABP1_BOVIN Polyadenylate-binding protein 1 OS=Bos taurus GN=PABPC1 PE=2 SV=1MNPSAPSYPMASLYVGDLHPDVTEAMLYEKFSPAGPILSIRVCRDMITRRSLGYAYVNFQQPADAERALDTMNFDVIKGKPVRIMWSQRDPSLRKSGVGNIFIKNLDKSIDNKALYDTFSAFGNILSCKVVCDENGSKGYGFVHFETQEAAERAIEKMNGMLLNDRKVFVGRFKSRKEREAELGARAKEFTNVYIKNFGEDMDDERLKDLFGKFGPALSVKVMTDESGKSKGFGFVSFERHEDAQKAVDEMNGKELNGKQIYVGRAQKKVERQTELKRKFEQMKQDRITRYQGVNLYVKNLDDGIDDERLRKEFSPFGTITSAKVMMEGGRSKGFGFVCFSSPEEATKAVTEMNGRIVATKPLYVALAQRKEERQAHLTNQYMQRMASVRAVPNPVINPYQPAPPSGYFMAAIPQTQNRAAYYPPSQIAQLRPSPRWTAQGARPHPFQNMPGAIRPAAPRPPFSTMRPASSQVPRVMSTQRVANTSTQTMGPRPAAAAAAATPAVRTVPQYKYAAGVRNPQQHLNAQPQVTMQQPAVHVQGQEPLTASMLASAPPQEQKQMLGERLFPLIQAMHPTLAGKITGMLLEIDNSELLHMLESPESLRSKVDEAVAVLQAHQAKEAAQKAVNSATGVPTV>sp|Q13310|PABP4_HUMAN Polyadenylate-binding protein 4 OS=Homo sapiens GN=PABPC4 PE=1 SV=1MNAAASSYPMASLYVGDLHSDVTEAMLYEKFSPAGPVLSIRVCRDMITRRSLGYAYVNFQQPADAERALDTMNFDVIKGKPIRIMWSQRDPSLRKSGVGNVFIKNLDKSIDNKALYDTFSAFGNILSCKVVCDENGSKGYAFVHFETQEAADKAIEKMNGMLLNDRKVFVGRFKSRKEREAELGAKAKEFTNVYIKNFGEEVDDESLKELFSQFGKTLSVKVMRDPNGKSKGFGFVSYEKHEDANKAVEEMNGKEISGKIIFVGRAQKKVERQAELKRKFEQLKQERISRYQGVNLYIKNLDDTIDDEKLRKEFSPFGSITSAKVMLEDGRSKGFGFVCFSSPEEATKAVTEMNGRIVGSKPLYVALAQRKEERKAHLTNQYMQRVAGMRALPANAILNQFQPAAGGYFVPAVPQAQGRPPYYTPNQLAQMRPNPRWQQGGRPQGFQGMPSAIRQSGPRPTLRHLAPTGSECPDRLAMDFGGAGAAQQGLTDSCQSGGVPTAVQNLAPRAAVAAAAPRAVAPYKYASSVRSPHPAIQPLQAPQPAVHVQGQEPLTASMLAAAPPQEQKQMLGERLFPLIQTMHSNLAGKITGMLLEIDNSELLHMLESPESLRSKVDEAVAVLQAHHAKKEAAQKVGAVAAATS>sp|Q5R8F7|PABP1_PONAB Polyadenylate-binding protein 1 OS=Pongo abelii GN=PABPC1 PE=2 SV=1MNPSAPSYPMASLYVGDLHPDATEAMLYEKFSPAGPILSIRVCRDMITRRSLGYAYVNFQQPADAERALDTMNFDVIKGKPVRIMWSQRDPSLRKSGVGNIFIKNLDKSIDNKALYDTFSAFGNILSCKVVCDENGSKGYGFVHFETQEAAERAIEKMNGMLLNDRKVFVGRFKSRKEREAELGARAKEFTNVYIKNFGEDMDDERLKDLFGKFGPALSVKVMTDESGKSKGFGFVSFERHEDAQKAVDEMNGKELNGKQIYVGRAQKKVERQTELKRKFEQMKQDRITRYQGVNLYVKNLDDGIDDERLRKEFSPFGTITSAKVMMEGGRSKGFGFVCFSSPEEATKAVTEMNGRIVATKPLYVALAQRKEERQAHLTNQYMQRMASVRAVPNPVINPYQPAPPSGYFMAAIPQTQNRAAYYPPSQIAQLRPSPRWTAQGARPHPFQNMPGAIRPAAPRPPFSTMRPASSQVPRVMSTQRVANTSTQTMGPRPAAAAAAATPAVRTVPQYKYAAGVRNPQQHLNAQPQVTMQQPAVHVQGQEPLTASMLASAPPQEQKQMLGERLFPLIQAMHPTLAGKITGMLLEIDNSELLHMLESPESLRSKVDEAVAVLQAHQAKEAAQKAVNSATGVPTV>sp|Q6DEY7|EPAB_XENTR Embryonic polyadenylate-binding protein OS=Xenopus tropicalis GN=epabp PE=2 SV=1MNATGAGYPLASLYVGDLHPDVTEAMLYEKFSPAGPIMSIRVCRDIATRRSLGYAYINFQQPADAERALDTMNFEVIKGRPIRIMWSQRDPGLRKSGVGNVFIKNLDESIDNKALYDTFSAFGNILSCKVVCDEHGSRGYGFVHFETQEAANRAIQTMNGMLLNDRKVFVGHFKSRRERELEYGAKVMEFTNVYIKNFGEDMDDKRLREIFSAFGNTLSVKVMMDDTGRSRGFGFVNYGNHEEAQKAVSEMNGKEVNGRMIYVGRAQKRIERQGELKRKFEQIKQERINRYQGVNLYVKNLDDGIDDDRLRKEFSPYGTITSAKVMTEGGHSKGFGFVCFSSPEEATKAVTEMNGRIVSTKPLYVALAQRKEERKAILTNQYMQRLATMRAMPGPLLGSFQQPANYFLPAMPQPPNRTFYSPNPVAPVRQAPQWTSHQSRPPQYQPPAPLMRAVPPRRMSSNISTMKQASTQVPRVAPHSQRVANIGTQTAGARAQVNPSIMRTMPHYKYSCAVRNVQPIGTNTHLQQVMEPAVLMQGQEPLTASSLASAPPQEQKQMLGERLYPLIHEMHPTLAGKITGMLLEIDNSELLHMLESPESLHSKVEEAVAVLQAHQAKENSQKSAQQSLI>sp|Q98SP8|EPABA_XENLA Embryonic polyadenylate-binding protein A OS=Xenopus laevis GN=epabp-a PE=1 SV=2MNATGAGYPLASLYIGDLHPDVTEAMLYEKFSPAGPIMSIRVCRDIATRRSLSYAYINFQQPADAERALDTMNFEVIKGRPIRIMWSQRDPGLRKSGVGNVFIKNLDESIDNKALYDTFSAFGNILSCKVVCDEHGSRGYGFVHFETHEAANRAIQTMNGMLLNDRKVFVGHFKSRRERELEYGAKVMEFTNVYIKNFGEDMDDKRLREIFSAFGNTLSVKVMMDDSGRSRGFGFVNYGNHEEAQKAVSEMNGKEVNGRMIYVGRAQKRIERQSELKRKFEQIKQERINRYQGVNLYVKNLDDGIDDDRLRKEFLPYGTITSAKVMTEGGHSKGFGFVCFSSPEEATKAVTEMNGRIVSTKPLYVALAQRKEERKAILTNQYMQRLATMRAMPGPLLGSFQQPANYFLSAMPQPPNRTFYSPNPVAPVRPAPQWASHQSRPPQYQPPTPLMRAVQPRRMSSNISTMKQASTQVPRVAQHSQRVANIGTQTAGARAQVNPSMMRTMPHYKYSCGVRNVQPIVSSTHLQQVMEPAVLMQGQEPLTASLLAGAPPQEQKQMLGERIYPVIHEMHPTLAGKITGMLLEIDNSELLHMLESPESLHSKVEEAVAVLQAHQAKENAQKSAQPSLI>sp|Q6GR16|EPABB_XENLA Embryonic polyadenylate-binding protein B OS=Xenopus laevis GN=epabp-b PE=2 SV=1MNATRAEYPLASLYIGDLHPDVTEAMLYEKFSPAGPIMSIRVCRDIATRRSLGYAYINFQQPADAERALDTMNFEVIKGRPIRIMWSQRDPGLRKSGVGNVFIKNLDDSIDNKALYDTFSAFGDILSCKVVCDEYGSRGYGFVHFETQEAANRAIQTMNGMLLNDRKVFVGHFKSRRERELEYGAKVMEFTNVYIKNFGEDMDDKRLKEIFSAFGNTLSVKVMMDNSGRSRGFGFVNYGNHEEAQKAVTEMNGKEVNGRMVYVGRAQKRIERQGELKRKFEQIKQERINRYQGVNLYVKNLDDGIDDDRLRKEFSPYGTITSTKVMTEGGHSKGFGFVCFSSPEEATKAVTEMNGRIVSTKPLYVALAQRKEERKAILTNQYMQRLATMRAMPGPLLGSFQQPANYFLPTMPQPSNRAFYSPNPVAPVRPAPQWASHQSRPPQYQPPAPLMRAVPPRRMHSNISTMKQASTQVPRVPLQSQRVANIGTQTAGARAQVNASIMRAMPHYKYSCGVRNVQPIGSSAHLQQVLEPAVLMQGQEPLTASLLAAAPLQEQKQILGERIYPLIHEMHPTLAGKITGMLLEIDNSELLHMLESPESLHSKVEEAVAVLQAHQAKESAPKSAPQSLI>sp|Q4VXU2|PAP1L_HUMAN Polyadenylate-binding protein 1-like OS=Homo sapiens GN=PABPC1L PE=2 SV=1MNASGSGYPLASLYVGDLHPDVTEAMLYEKFSPAGPILSIRVCRDVATRRSLGYAYINFQQPADAERALDTMNFEMLKGQPIRIMWSQRDPGLRKSGVGNIFIKNLEDSIDNKALYDTFSTFGNILSCKVACDEHGSRGFGFVHFETHEAAQQAINTMNGMLLNDRKVFVGHFKSRREREAELGARALEFTNIYVKNLPVDVDEQGLQDLFSQFGKMLSVKVMRDNSGHSRCFGFVNFEKHEEAQKAVVHMNGKEVSGRLLYAGRAQKRVERQNELKRRFEQMKQDRLRRYQGVNLYVKNLDDSIDDDKLRKEFSPYGVITSAKVMTEGGHSKGFGFVCFSSPEEATKAVTEMNGRIVGTKPLYVALAQRKEERKAILTNQYMQRLSTMRTLSNPLLGSFQQPSSYFLPAMPQPPAQAAYYGCGPVTPTQPAPRWTSQPPRPSCASMVRPPVVPRRPPAHISSVRQASTQVPRTVPHTQRVANIGTQTTGPSGVGCCTPGRPLLPCKCSSAAHSTYRVQEPAVHIPGQEPLTASMLAAAPLHEQKQMIGERLYPLIHDVHTQLAGKITGMLLEIDNSELLLMLESPESLHAKIDEAVAVLQAHQAMEQPKAYMH>sp|P21187|PABP_DROME Polyadenylate-binding protein OS=Drosophila melanogaster GN=pAbp PE=1 SV=3MASLYVGDLPQDVNESGLFDKFSSAGPVLSIRVCRDVITRRSLGYAYVNFQQPADAERALDTMNFDLVRNKPIRIMWSQRDPSLRRSGVGNVFIKNLDRAIDNKAIYDTFSAFGNILSCKVATDEKGNSKGYGFVHFETEEAANTSIDKVNGMLLNGKKVYVGKFIPRKEREKELGEKAKLFTNVYVKNFTEDFDDEKLKEFFEPYGKITSYKVMSKEDGKSKGFGFVAFETTEAAEAAVQALNGKDMGEGKSLYVARAQKKAERQQELKRKFEELKQKRHESVFGVNLYVKNLDDTIDDDRLRIAFSPYGNITSAKVMTDEEGRSKGFGFVCFNAASEATCAVTELNGRVVGSKPLYVALAQRKEERKAHLASQYMRHMTGMRMQQLGQIYQPNAASGFFVPTLPSNQRFFGSQVATQMRNTPRWVPQVRPPAAIQGVQAGAAAAGGFQGTAGAVPTQFRSAAAGARGAQPQVQGTHAAAAAANNMRNTGARAITGQQTAAPNMQIPGAQIAGGAQQRTSNYKYTSNMRNPPVPQLHQTQPIPQQLQGKNSEKLIASLLANAKPQEQKQILGERLYPMIEHMHANLAGKITGMLLEIENSELLHMIEDQEALKAKVEEAVAVLQVHRVTEPAN>sp|P20965|PABPA_XENLA Polyadenylate-binding protein 1-A OS=Xenopus laevis GN=pabpc1-a PE=1 SV=3MNPSAPSYPMASLYVGDLHQDVTEAMLYEKFSPAGPILSIRVCRDMITRRSLGYAYVNFQQPADAERALDTMNFDVIKGRPVRIMWSQRDPSLRKSGVGNIFIKNLDKSIDNKALYDTFSAFGNILSCKVVCDENGSKGYGFVHFETQEAAERAIDKMNGMLLNDRKVFVGRFKSRKEREAELGARAKEFTNVYIKNFGDDMNDERLKEMFGKYGPALSVKVMTDDNGKSKGFGFVSFERHEDAQKAVDEMNGKDMNGKSMFVGRAQKKVERQTELKRKFEQMKQDRITRYQGVNLYVKNLDDGIDDERLRKEFLPFGTITSAKVMMEGGRSKGFGFVCFSSPEEATKAVTEMNGRIVATKPLYVALAQRKEERQAHLTNQYMQRMASVRVPNPVINPYQPPPSSYFMAAIPPAQNRAAYYPPGQIAQLRPSPRWTAQGARPHPFQNMPGAIRPTAPRPPTFSTMRPASNQVPRVMSAQRVANTSTQTMGPRPTTAAAAAASAVRAVPQYKYAAGVRNQQHLNTQPQVAMQQPAVHVQGQEPLTASMLAAAPPQEQKQMLGERLFPLIQAMHPTLAGKITGMLLEIDNSELLHMLESPESLRSKVDEAVAVLQAHQAKEAAQKVVNATGVPTA>sp|Q6IP09|PABPB_XENLA Polyadenylate-binding protein 1-B OS=Xenopus laevis GN=pabpc1-b PE=2 SV=1MNPSAPSYPMASLYVGDLHPDVTEAMLYEKFSPAGPILSIRVCRDMITRRSLGYAYVNFQQPADAERALDTMNFDVIKGKPVRIMWSQRDPSLRKSGVGNIFIKNLDKSIDNKALYDTFSAFGNILSCKVVCDENGSKGYGFVHFETQEAAERAIDKMNGMLLNDRKVFVGRFKSRKEREAELGARAKEFTNVYIKNFGEDMDDERLKEWFGQYGAALSVKVMTDDHGKSRGFGFVSFERHEDAQKAVDDMNGKDLNGKAIFVGRAQKKVERQTELKRKFEQMKQDRITRYQGVNLYVKNLDDGIDDERLRKEFTPFGSITSAKVMMEGGRSKGFGFVCFSSPEEATKAVTEMNGRIVATKPLYVALAQRKEERQAHLTNQYMQRMASVRVPNPVINPYQPPPSSYFMAAIPPAQNRAAYYPPGQIAQLRPSPRWTAQGARPHPFQNMAGAIRPSAPRPPTFSTMRPTSQVPRVMSAQRVANTSTQTMGPRPTTAAAAATSAVRTVPQYKYAAGVRNTQQHLNTQPQVAMQQPAVHVQGQEPLTASMLAAAPPQEQKQMLGERLFPLIQAMHPTLAGKITGMLLEIDNSELLHMLESPESLRSKVDEAVAVLQAHQAKEAAQKVVSATGVPTA
